# Suicidality among gender minorities in Karnataka, South India

**DOI:** 10.1186/s12888-021-03043-2

**Published:** 2021-01-11

**Authors:** Shiva S. Halli, Shajy Isac, Parinita Bhattacharjee, Sumit Dutta, B. M. Ramesh, Robert Lorway, James Blanchard

**Affiliations:** 1 Institute for Global Public Health, Department of Community Health Sciences, Rady Faculty of Health Sciences, University of Manitoba, Winnipeg, Manitoba R3E 0T6 Canada; 2 India Health Action Trust, E-37, Defense Colony, New Delhi, 560017 India; 3 Adobe Systems Pvt. Ltd, Block A, Prestige Platina Tech Park, Marthahalli, Sarjapur Road, Bengaluru, Karnataka 560087 India

**Keywords:** Suicidality, Gender minorities, *Hijra* and *Kothi*, Anxiety, Depression, Karnataka, India

## Abstract

**Background:**

It is argued that Indian gender minorities displayed differential mental health problems and suicide attempts. Hence, the study was intended to understand the prevalence of anxiety, depression and suicidality among this group, specifically those living in a metropolitan city in South India.

**Methods:**

The data was generated from a cross-sectional study that employed a structured questionnaire to collect information about experiences of anxiety, depression and suicidal behavior among gender-diverse individuals in Bangalore, the capital city of Karnataka state. The study used stratified simple random sampling of eligible individuals who were 18 years of age and older and who were enrolled in an HIV prevention program implemented for gender-diverse individuals run by the Karnataka Health Promotion Trust and the University of Manitoba at the time of the study (2012). Bivariate and multivariate analyses were used to assess the relative contribution of various factors that affect suicide ideation or actual attempts among the gender diverse participants.

**Results:**

Results showed that 62% whose main source of income was *Basti* (socially sanctioned practice of begging)*,* 52% of *Hijras,* 56% who lived with their Gurus, 58% who were not happy with their physical appearance, 55% who consumed alcohol daily, and 63% who experienced high depression had ever thought of or attempted suicide in the month prior to the survey. However, multivariate analysis showed that respondents who were not happy with their physical appearance and thought of changing it had significantly higher odds (AOR = 2.861; CI 1.468,5.576; *p* = 0.002) of either having thoughts that it was better being dead or wished they died. Similarly, those who experienced high depression, their odds of either having had thoughts of or having attempted suicide increased by three times (AOR = 3.997; CI 1.976, 8.071; *p* < 0.000).

**Conclusions:**

It is observed that a high percentage of gender minorities had attempted suicide or thought of suicide during the month preceding the data collection. The findings bring new insights on the proximate determinant of physical appearance on the suicidality of gender minorities assigned male at birth and appropriate to account for this while addressing the mental health issues.

**Supplementary Information:**

The online version contains supplementary material available at 10.1186/s12888-021-03043-2.

## Background

In India, suicide deaths are much higher than the global prevalence. The most recent age standardized suicide death rate for India is 21.2 deaths per 100,000 compared to the corresponding global prevalence, which is almost half—11 deaths per 100,000 [1]. Despite higher rates of suicide in the Indian general population, to date there remains a lack of suicide prevention policies and programs at the state or central government level. However, toward the goal of planning and implementing suicide prevention programs in India, a nationally representative population-based study on suicidality was more recently conducted. It was found that for every suicide death there was more than 200 people with suicidality as well as more than 15 suicide attempts [2].

Geographical heterogeneity in suicide death rates have been found in India [1], with the highest rates of suicide found in southern states and the lowest rates found in northern states [3]. Significant variations by sex were found with males having higher suicide rates that decreased less overtime (between 2001 and 2013) compared to females. Furthermore, females in the age group of 15–29 years were found to have the highest suicide rate, while males in the age group of 45–49 had the highest suicide rate [3]. Furthermore, male suicide rates were found to be higher in regions of India with higher unemployment levels. For both females and males, higher suicide rates were found in regions with greater development, pointing to the possibility of modernization as contributing toward suicide risk [3]. Regarding other socio-economic determinants of suicide in India, different rates of suicide have been identified among religious groups. Overall, the highest suicide rates were found among Christians while the lowest rates were found among Muslims and Sikhs [4]. However, this picture changes substantially according to geography: although Muslims and Sikhs generally had lower suicide rates than Christians and Hindus, this was not the case in Rajasthan and Orissa and other states in central India [4].

A review of the literature also revealed that in many parts of the world, among other minorities, such as the transgender individuals, rates of suicide attempts are substantially higher compared to the general population [5]. Although there is a lack of scientific documentation of suicide rates or suicide attempts among transgender persons in India, a report in Karnataka state showed that the suicide rate among transgender individuals is approximately 31% [6]. Moreover, the report suggests that 50% of transgender teens as well as middle-aged transgender adults had attempted suicide at least once [6].

Gender is a complex aspect of human social identity that is often viewed along a spectrum between what is socially constructed as masculine or feminine, which may be tied to notions of maleness and femaleness or altogether dislodged from notions of anatomical or genetic sex [7]. What constitutes masculinity and femininity indeed varies throughout the world, shaping a person’s internal sense of being masculine, feminine, or non**-**binary, in culturally specific ways [7].

Among the two gender nonconforming groups considered in the paper, the term *Hijra* (self-identified) is a culturally accepted gender identity category employed throughout South Asia, and since ancient times [8]. It refers to those assigned at birth as male but who later identify in gender and sexual nonconforming terms, as “neither man nor woman”, “neither male nor female” or “third gender”. In recent decades, this term is employed interchangeably with the western gender identity category “transgender woman”. The second group, who are often identified in the public health literature as “men who have sex with men” and self-identify with the term *Kothi*, similarly engage in gender nonconformity in terms of behavior and attire. However, they often avoid expressing their gender difference publically because of fear of stigma and discrimination [9, 10]. Many are unable to reconcile their gender identity and the social role that they are expected to play, leading to feelings of alienation [11].

While lesbian, gay bisexual, transgender and queer social movements have begun to flourish in many parts of the World, until recently the colonial law in India (Section 377 of the Penal Code Act XLV of 1860) has criminalized homosexuality [5]. In the 1990s, India did see important socio-political shifts as an array of human rights programs and interventions led by various sexual and gender minority rights organizations mushroomed across many states of India. Further mobilizations of gender and sexual minorities took place in the era of HIV interventions, especially in the first decade of 2000s and with the deployment of the multi-state, Bill & Melinda Gates-funded HIV initiative known as Avahan. These HIV programs indeed hinged on the engagement of gender and sexual minority-led organizations, which reinforced social movements and stirred protestation demanding social change. Despite these mobilizations, interventions, and social movements, gender and sexual minorities in India, however, only recently have legal protections been afforded when: (1) homosexuality was decriminalized in 2018 (i.e., when Section 377 of the Penal Code was declared unconstitutional) and (2) the Transgender Protection of Rights Bill was finally passed by the parliament in 2019.

Despite these seemingly dramatic political shifts, social stigmatization continues to be pervasive in Indian society. Indeed, sexual and gender minorities are subject to a great deal of prejudice, social stress, social exclusion, violence and hatred [12, 13]. Many internalize a sense of shame around their sexual orientation and sense of gender difference [12, 14]. According to researchers, these negative consequences are collectively experienced as minority stress, which refers not only to the stress of being discriminated against, but includes one’s internalization of these negative beliefs [12]. There is ample evidence that these beliefs are associated with psychological distress and mental disorders [13, 15, 16]. In this regard, minority stress has been suggested as an important determinant of mental health outcomes among sexual and gender minorities [12, 13]. In a recent meta-analysis, King and colleagues concluded that the risk for lifetime mood, anxiety and substance use disorders was at least 1.5 times higher in lesbian, gay and bisexual people [15]. In the case of India, to the best of authors’ knowledge, an exact prevalence of suicide or suicidal attempt among *Kothis* and *Hijras* is not available. However, as indicated earlier, an unpublished report suggests that 31% of the *Hijras* in India commit suicide and half of them have attempted suicide at least once before their 20th birthday [6].

Suicide is a complex behavior which results from the complicated interaction of socio-cultural, psychological, cognitive, and environmental factors. In a highly patriarchal society such as India, instead of an approach that is inclusive of gender and sexual diversity, rejection and lack of familial and social support, gender dysphoria associated with extreme stressful experiences, and lack of legal measures for protection are some of the factors with which transgender *Hijras* and gender non-conforming *Kothis* must contend [5, 13]. Indian society in many ways still portrays gender and sexual minorities as though they are socially deviant in violation of social norms and values. Consequently, they continue to be subjected to ridicule, discrimination and harassment by others including family members, parents and friends [13]. Furthermore, they are deprived of education and livelihood opportunities which can undermine their self-esteem, and precipitate forms of depression and suicidal behavior and ideation.

Studies conducted in the United States, Australia and England have documented, respectively, that 41, 50 and 48% of transgender persons attempted suicide at least once in their lives [5, 10]. Although several studies in India estimate and explain suicide prevalence rates at the national and regional levels and analyze suicidality and its correlates for the general population to help policy makers [1–3, and], to the best of the authors’ knowledge no scientifically sound study concerning suicide and suicidality has been conducted among gender minority groups in India, despite their global vulnerability. This could be largely due to the marginalized status of transgender people, reinforced by conditions of homelessness and poverty, and their inability to access social services and other social welfare entitlements. However, the Supreme Court ruling of 2014, which moved to protect the social liberties of Indian citizens regardless of gender identity [17], and the passing of transgender rights and protections legislation in 2016 has created a more receptive policy climate to take action on scientific evidence pertaining to transgender people. Although our data set is 8 years old, given the existence of only limited evidence our study takes an important step in filling a significant gap in the literature and advancing evidence-based policy making for one of the most highly disadvantaged groups in India.

Although suicidal behavior has been linked to sexual minority status in previous studies [18], the current study focuses on the relationship between suicidal behavior and *gender* minority status, examining this among transgender women and gender nonconforming men (i.e., gender minorities). Mental disorder has been shown as a strong risk factor for suicidal behavior in general, and it is important to consider this when examining suicide attempts among gender minorities. High rates of suicidal behavior reported in previous work may have been conflated by high rates of mental disorders in this population [19]. This increased likelihood is not simply an artefact of high rates of mental disorder in this group; rather, it could be independent of a psychiatric diagnosis at higher rates than in groups of cis-gender people.

It is possible that other factors not assessed earlier could be confounding the relationship between mental disorders and gender identity. Previous work exploring minority stress has indicated strong relationships between discrimination and a variety of mental health outcomes [9]. This study explores the experiences of gender minorities as a possible link between gender and the development of anxiety, depression and suicidal behavior. In addition to the novel aspects of this study, there are a number of key strengths that need to be emphasized. The study draws on an epidemiologic sample of gender minorities in Karnataka, India. The sample size allows the inclusion of two specific gender subtypes, transgender women and gender non-conforming men separately, a feature that has been noted as a significant methodological limitation in past work. It is argued that the gender minorities displayed differential mental disorders and suicide attempts. Thus, the study is intended to understand the prevalence of anxiety, depression and suicidal behavior and ideation and attempts among gender minorities with special reference to the role of physical appearance in the metropolitan city of Bangalore, in Karnataka state, South India.

## Design and methodology

The data is generated from a cross-sectional study that employed a structured questionnaire to collect information about experiences of anxiety, depression and suicidal behavior among gender-diverse individuals in Bangalore, Karnataka [20]. The study used stratified simple random sampling of eligible individuals who were 18 years of age and older and who were enrolled in the HIV prevention program implemented for gender-diverse individuals by the Karnataka Health Promotion Trust and the University of Manitoba at the time of the study (2012). Prospective participants were selected after stratifying them by gender subtype, *Hijra* and *Kothi*, which included individuals who were enrolled in the program, and who chose to self-identify [20]. Six interviewers were recruited from the *Hijra* and *Kothi* community based on their knowledge of the local language Kannada, rapport within the community, sensitivity, and knowledge of research ethics and norms of confidentiality. Interviewers were provided 6 days of intensive training to prepare for the data collection process that adhered to the ethical principles of conducting research on a sensitive subject [20]. In order to protect the privacy of study participants, the questionnaires were administered after obtaining informed written consent at one of the following locations: the drop-in center of the targeted HIV program, a *Hamam* (Bath House) where groups of *Hijras* commonly work, or the home of the study participant. The *Hijra* participants tended to choose a *Hamam* or their home as their preferred location for the interview whereas *Kothi* participants, many of whom lived with their families, preferred the drop-in center. A random sample of 282 individuals were interviewed individually in English or the local language, Kannada, by the trained peer interviewers using a structured questionnaire [20]. The Institutional Ethical Review Board of St. John’s Medical College in Bangalore, India, approved this study. Details of the study design, methods and measures used in this paper are described in detail in the survey report and subsequent study by the authors [13, 20].

### Measures

The information was collected on socio-demographic characteristics, perception of body image, relationships, alcohol consumption, health, anxiety, depression and suicidality. Socio-demographic variables included gender identity, age, religion, education level, main income source, history of being married to a woman, and living arrangement [20]. Gender diverse individuals’ minority status was based on self-identified gender identity only. The main outcome variable for the study was “Have you ever thought or attempted suicide in the past one month”. The survey asked two questions on ‘thought’ or ‘attempted’ suicide in the past month. The questions were “In the past month, did you think about suicide” and “In past one month have you attempted suicide or to harm yourself?” We computed the outcome variable if the answer to either of the question was ‘Yes’ to 1 and if neither was ‘Yes’ to 0. There was also a question about perception of body image: “Is there anything you would like to change about your physical appearance?” The questions that were focused on aspects related to depression and anxiety were derived from the World Health Organization’s Quality of Life Scale (WHOQOLHIV) [21], the Hamilton Anxiety Scale (HAM-A), [22] and the Beck Depression Inventory (BDI-II) [23]. A formative qualitative contextualization process based on prior focus group discussions and in-depth interviews with community members was used to select a set of questions from the three standard scales, grounding the questionnaire in the current sociopolitical realities within the community. The composite score was used separately for depression and anxiety, wherein we used 8 questions of a maximum score of 16 to arrive at the composite score of depression. Factor analysis was conducted on this series of questions to identify a set of questions that could be used to calculate a composite depression score measuring symptoms of depression for participants. The Cronbach’s alpha for the resulting scoring system was 0.671. A composite depression score was calculated by adding together the number of selected questions to which the participant indicated a “yes” response, with a maximum possible value of 16. There were two questions asked to assess the anxiety level of participants, which were used to derive the score for anxiety with a maximum score of 4. The score for anxiety was computed as “high anxiety” if the answers to both questions were “always” or at least one was “always” and the other answer was ‘sometimes’; otherwise, it was computed as “low/medium anxiety”. The depression and anxiety scores are not a diagnosis and instead provides a means of measuring the degree to which study participants experienced depressive symptoms and feelings of anxiety. The complete questionnaire is available in the published report [20].

### Data analysis

The data were transferred into SPSS format from CSPro and data analysis was carried out in SPSS25.0 in two stages. First, bivariate analysis was performed for all the indicators of dependent variables such as anxiety, depression and suicide thought or attempt by independent variables. Chi-square test of significance was applied in order to identify the significant associations between these variables.

In the second level of the analysis, multivariate logistic regression methods were used to assess the relative contribution of various factors that affect suicide thought or attempt among the gender diverse individuals. Based on the findings of the bivariate analyses, the combined effect of these predictors were assessed using multivariate logistic regression technique in order to identity possible significant predictors of suicide thought or attempt among gender minorities.

## Results

Of the total respondents (282), about 48% were below the age of 30 years, 18% had little or no formal education, 86% were Hindus, 18% were into sex work, 74% were never married, 49% were living with family or spouse, 62% identified as *Kothis*, 38% identified as *Hijras*, and 51% were migrants (Additional file 1). Table 1 shows that, of the total respondents, about 45 and 33% of the respondents reported that they were experiencing high anxiety and high depression respectively. Table 1 also presents bi-variate associations between background characteristics with level of anxiety and depression. The only significant factors associated with anxiety were education and alcohol consumption; 66% of respondents with little or no formal education were experiencing high anxiety as compared to 43% of the respondents with 10+ years of schooling. Further, 57% of the respondents who consumed alcohol daily suffered from high anxiety as compared to 42% among those consumed never/less often. Similarly, the only significant factors associated with depression were education and marital status; 60% of respondents with little or no formal education compared to 28% with10+ years of schooling and 53% of the respondents who were married but currently not living with their spouse compared to 30–32% of never married or currently living with spouse suffered from high depression.

**Table 1:** Percent of participants experienced anxiety and depression by background characteristics

Characteristics	Total	Overall anxiety			Overall depression		
		Low/medium	High	p	Low/medium	High	p
Total	282	54.6	45.4		67.5	32.5	
**Age of the respondents**							
< 30 years	135	56.3	43.7	0.586	65.6	34.4	0.523
30+ years	147	53.1	46.9		69.3	30.7	
**Education level**							
Little or no formal education	50	34.0	66.0	0.003	40.4	59.6	0.000
5–9 years schooling	67	64.2	35.8		75.4	24.6	
10+ years schooling	165	57.0	43.0		72.3	27.7	
**Main source of income**							
Service/business/trade	104	58.7	41.3	0.073	64.6	35.4	0.866
Basti	63	63.5	36.5		68.3	31.7	
Sex work	51	41.2	58.8		68.0	32.0	
Others	64	50.0	50.0		71.0	29.0	
**Religion**							
Hindu	241	52.7	47.3	0.118	66.7	33.3	0.467
Others	41	65.9	34.1		72.5	27.5	
**Personal identity**							
*Kothi*	174	55.7	44.3	0.626	66.5	33.5	0.637
*Hijra*	108	52.8	47.2		69.2	30.8	
**Current marital status**							
Currently living with spouse	41	46.3	53.7	0.394	68.3	31.7	0.034
Ever married, but currently not living with spouse	32	50.0	50.0		46.7	53.3	
Never married	209	56.9	43.1		70.5	29.5	
**Living status**							
Living with family/spouse	137	54.0	46.0	0.400	66.9	33.1	0.068
Living alone/with a male partner	111	58.6	41.4		73.6	26.4	
Living with guru	33	45.5	54.5		51.6	48.4	
**Status of living in the city**							
Since birth	132	54.5	45.5	0.829	69.5	30.5	0.314
Migrated to the city	139	53.2	46.8		63.6	36.4	
**Like to change physical appearance**							
No	194	56.2	43.8	0.430	69.4	30.6	0.342
Yes	88	51.1	48.9		63.5	36.5	
**Alcohol consumption**							
Never/Less often	217	58.1	41.9	0.033	69.7	30.3	0.163
Everyday	65	43.1	56.9		60.3	39.7	

The results in Table 2 revealed that about two-fifth (42%) of the respondents thought in the past month it was better being dead or wished they died. In case of bi-variate associations between background characteristics with those who thought in the past month it was better being dead or wished they died, significant factors were source of income, personal identity, living arrangement, physical appearance, consumption of alcohol and depression. To be specific, 62% whose main source of income was *Basti* (socially sanctioned practice of begging)*,* 52% of *Hijras,* 56% who were living with their Gurus, 58% who were not happy with their physical appearance, 55% who consumed alcohol daily, and 63% who experienced from high depression thought of or attempted suicide in the month prior to the survey.

**Table 2:** Percent participants either thought or attempted suicide in the past month by background characteristics

Characteristics	Total	Percent	p
Total	278	42.3	
**Age of the respondents**			
< 30 years	132	45.9	0.249
30+ years	146	39.0	
**Education level**			
Little or no formal education	50	46.0	0.143
5–9 years schooling	65	31.8	
10+ years schooling	163	45.4	
**Main source of income**			
Service/business/trade	104	44.2	0.000
Basti	63	61.9	
Sex work	49	38.8	
Others	62	22.2	
**Religion**			
Hindu	239	42.3	0.977
Others	39	42.5	
**Personal identity**			
*Kothi*	171	36.0	0.007
*Hijra*	107	52.3	
**Current marital status**			
Currently living with spouse	41	29.3	0.067
Ever married, but currently not living with spouse	32	56.3	
Never married	205	42.7	
**Living arrangement**			
Living with family/spouse	134	34.1	0.022
Living alone/with a male partner	111	47.7	
Living with guru	32	56.3	
**Status of living in the city**			
Since birth	129	37.7	0.056
Migrated to the city	138	49.3	
**Like to change physical appearance**			
No	193	35.2	0.000
Yes	85	58.1	
**Alcohol consumption**			
Never/Less often	213	38.3	0.015
Everyday	65	55.4	
**Overall anxiety**			
Low/medium	152	37.5	0.076
High	126	48.0	
**Overall depression**			
Low/medium	181	32.6	0.000
High	86	63.2	

The bivariate and multivariate odds ratios of logistic regression to understand the respondents who thought it was better being dead or wished they died 1 month prior to the survey are presented in Tables 3. The bivariate analysis shows that being a *Hijra*, wanting to change physical appearance, overall depression and anxiety as significant predictors of those who thought or attempted suicide. However, after controlling for the background characteristics in multivariate analysis, personal identity and anxiety were insignificant, at 5% level of significance. On the other hand, respondents who were not happy with their physical appearance and thought of changing it have significantly higher odds (AOR = 2.861; CI 1.468, 5.576; *p* = 0.002) of either thought it was better being dead or wished they died. Similarly, those who were experiencing high depression, their odds of either thought or attempted suicide increased by three times (AOR = 3.997; CI 1.976, 8.071; *p* < 0.000).

**Table 3:** Multivariate analysis of predictors of mental health as measured by thought or attempted suicide in the past month

		Bivariate		Multivariate	
		Exp(B) [95% C.I]	p	Exp(B) [95% C.I]	p
Personal Identity	*Kothi*	1.000		1.000	
	*Hijra*	1.948 [1.193, 3.182]	0.008	2.281 [0.772, 6.739]	0.136
Desire to change physical appearance	No	1.000		1.000	
	Yes	2.553 [1.517, 4.296]	0.000	2.861 [1.468, 5.576]	0.002
Overall depression	Low/medium	1.000		1.000	
	High	3.554 [2.081, 6.070]	0.000	3.997 [1.976, 8.071]	0.000
Overall anxiety	Low/medium	1.000		1.000	
	High	1.540 [0.955, 2.486]	0.077	0.808 [0.416, 1.572]	0.531

The multivariate model controls for main source of income, marital and living status, status of living in the city and alcohol consumption

## Discussion

Among transgender women known as *Hijras* and gender nonconforming men known as *Kothis* who participated in this study who claim an identity as either a *Hijra* or *Kothi*, 45% of them experienced high anxiety, one third reported a high level of depression and 42% of them thought of attempting suicide or attempted suicide 1 month preceding to the survey. Those who experienced high anxiety were respondents with no formal education and who consumed alcohol daily. Similarly, those who experienced high depression were respondents with little or no formal education and who were married but not living with their spouse. After adjusting for the background characteristics, the only significant predictors which increased the odds of either suicidal thought or attempted suicide 1 month preceding to the survey were wanting to change physical appearance and depression. India is a patriarchal society and certain diversities, which do not conform to patriarchal norms, are often rejected. The varying degree of stigma and discrimination towards gender minorities is rooted in political, economic and ideological structures [24, 25]. Therefore, transgender women and non-conforming men are faced with stigma and discrimination within the family and society. Such forms of widespread social discrimination could foster internalized transphobia and stigma within gender minorities. This self-stigma undermines the mental health of that individual and may inhibit seeking professional help due to fear of identity disclosure [26]. Also, experiences of stigma and discrimination lead to greater vulnerabilities among the gender minorities thereby resulting in high levels of depressive symptoms, anxiety and higher rates of psychiatric disorders [27]. Consequently, these minorities possess greater risk of attempted suicides [19, 28].

After controlling for anxiety and depression, wanting to change physical appearance being one of the important proximate determinants of suicidal behavior among the gender minorities may be difficult to comprehend. However, it can be argued that these individuals are faced with two possible alternatives: (1) to live by openly embracing and expressing their gender nonconformity, only to experience relentless negative social feedback or (2) to conceal their gender nonconformity to adhere to larger social norms, to be socially accepted. Both possibilities here are, of course, highly influenced by personal, psychological and group norms. Wider societal norms, however, are largely shaped by patriarchal cultural values that assign masculine-gender norms to “male bodies”, to uphold its system and maintain male privilege. It is possible that these differences are also dependent upon the context that the minorities are thought of in terms of their disadvantaged status. The disadvantaged status might have different consequences on different individuals. For instance, those individuals that experience greater socio-economic deprivation associated with lower social class may resist their desire to change their physical appearance and may live with lower quality of life in the face of perceived minority status. On the other hand, those individuals who have a strong desire to change their physical appearance from a more masculine to a more feminine one but unable to do so, due to their disadvantaged status, may be likely to attempt suicide or think of suicide. It is also found in other Indian states such as Maharashtra that those *Hijras/Kothis* unable to change their physical appearance suffer from feelings of shame, humiliation, internalized stigma and low self-worth [29].

Amid India’s socio-economic and cultural diversity, a disaggregated analysis of *Hijras/Kothis* suicidality is useful to realize that these groups may experience higher suicide rates compared to the general population. It is not to undermine the problem of suicide in the general population of India but to uncover and underscore the distinctive reality of gender minorities who suffer a sense of body dysmorphia and depression while lacking the dedicated services to address these critical issues. Hence, policy makers and programmers may need to develop innovative and supportive approaches to address the access to barriers to mental health services faced by *Hijras/Kothis*. Alongside research on suicide in the general population in India, this research can help sensitize policy makers to generate more inclusive policies that are more appropriately tailored to the distinctive needs of gender minorities.

### Limitations

The data used in the study is based on a cross-sectional design, and therefore, whether experiences of depression and desired body image indeed caused suicide attempts is difficult to determine. Future research should consider longitudinal studies or randomized control trials to establish clear causal links. The study also suffers from recall bias as the study subjects were asked to report experiences of suicidality up to 1 year prior to the study. Lastly, the study included a sample of gender minorities among those enrolled in an HIV program in Bangalore. In short, the pool of program attendants provided the sampling universe. The program enrolled and provided HIV services to those engaged in particular sexual practices. Therefore, this study refers to aspects of mental health among gender minorities who were engaged in particular HIV risk behaviors, who were highly vulnerable to HIV, and who were exposed to a health promotion program context. This study, therefore, does not represent all gender minorities in Bangalore, but only those who are most vulnerable to HIV infection. The caution should be exercised in generalizing the results to the larger gender minority population.

## Conclusions

In addition to noting the high percentage of gender minorities that have attempted suicide or thought of suicide during the month preceding the data collection process, the findings of the study also bring new insights on proximate determinants, such as physical appearance, and their relationship to attempted suicide or thoughts of suicide among gender minorities. To understand gender minorities’ suicidal behavior, it is useful to recognize that several approaches interact. There is a strong overlap among cultural norms, economic, social and psychological approaches. Failure to recognize this in the past research on gender minority status and suicidal behavior led to inconsistent conclusions. Regardless of the etiology, gender minorities are a population vulnerable to life-threatening mental health outcomes. Clinicians need to be aware of these negative mental health consequences when assessing gender minorities, and need to maintain a high level of attentiveness during suicide risk assessments and counseling. A study conducted among *Hijra* community in Lahore and Karachi, Pakistan, not only suggested psychological counseling to deal with low self-esteem and with the animosity directed toward gender minorities, but it also recognizes the importance of establishing community support networks to mediate access to medical care and social services [30]. At the same time, ongoing efforts to strengthen the protection of human rights for gender minorities are crucial for unseating the broader socio-political factors that feed stigmatization. For this reason, it is also hoped that these findings provide vital evidence to support advocacy efforts directed toward this social change making.

## Supplementary Information

**Additional file 1.** Socio-demographic profiles of respondents

**Additional file 2.** Baseline Survey Questionnaire for Psychosocial Support Project, 2011–12 Karnataka Health Promotion Trust, Bangalore

## Data Availability

The data that support the findings of this study are available from the Institute for Global Public Health, the University of Manitoba but restrictions apply to the availability of these data, which were used under license for the current study, and so are not publicly available. Data are however available from the authors upon reasonable request and with permission of the Institute for Global Public Health, the University of Manitoba, Winnipeg, Canada.
