# Supplementary material for: Suicidality among gender minorities in Karnataka, South India
Source: BMC Psychiatry. 2021 Jan 11;21:25. doi: 10.1186/s12888-021-03043-2 (PMC7798192; doi:10.1186/s12888-021-03043-2)
Supplement: Supplementary file 1 — Additional file 1. Socio-demographic profiles of respondents [file 12888_2021_3043_MOESM1_ESM.docx]

**Appendix 1: Socio-demographic profiles of respondents**

| Characteristics | Percent |
| --- | --- |
| Number | 282 |
| **Age of the respondents** |  |
| <30 years | 47.9 |
| 30+ years | 52.1 |
| Mean age | 31.6 |
| **Education level** |  |
| Little or no formal education | 17.7 |
| 5-9 years schooling | 23.8 |
| 10+ years schooling | 58.5 |
| **Main source of income** |  |
| Service/business/trade | 36.9 |
| Basti | 22.3 |
| Sex work | 18.1 |
| Others | 22.7 |
| **Religion** |  |
| Hindu | 85.5 |
| Others | 14.5 |
| **Personal identity** |  |
| *Kothi* | 61.7 |
| *Hijra* | 38.3 |
| **Current marital status** |  |
| Currently living with spouse | 14.5 |
| Ever married, but currently not living with spouse | 11.3 |
| Never married | 74.1 |
| **Living status** |  |
| Living with family/spouse | 48.8 |
| Living alone/with a male partner | 39.5 |
| Living with guru | 11.7 |
| **Status of living in the city** |  |
| Since birth | 48.7 |
| Migrated to the city | 51.3 |
