# Supplementary material for: Suicidality among gender minorities in Karnataka, South India
Source: BMC Psychiatry. 2021 Jan 11;21:25. doi: 10.1186/s12888-021-03043-2 (PMC7798192; doi:10.1186/s12888-021-03043-2)
Supplement: Supplementary file 2 — Additional file 2. Baseline Survey Questionnaire for Psychosocial Support Project, 2011–12 Karnataka Health Promotion Trust, Bangalore [file 12888_2021_3043_MOESM2_ESM.pdf]

Baseline Survey Questionnaire for Psychosocial Support Project, 2011-12
Karnataka Health Promotion Trust, Bangalore

SECTION I: INTERVIEW INFORMATION
ವಿಭಾಗ ೧: ಸಂದರ್ಶನ ಮಾಹಿತಿ

Study number/ಅಧ್ಯಯನ ಸಂಖ್ಯೆ.. [ ][ ][ ]

Zone/ಜೊನ್______________________________ [ ]

Type of Locale/ಸ್ಥಳ (Public garden/ಉದ್ಯಾನವನ=1, Public toilet/ಸಾರ್ವಜನಿಕ ಶೌಚಾಲಯ=2,
Railway station/ರೈಲ್ವೆಸ್ಟೇಷನ್=3, Bus standstop/ಬಸ್ ನಿಲ್ದಾಣ=4,
Hamam/Residential area/ಹಮಾಮ್/ವಾಸ ಸ್ಥಳ=5, Other/ಇತರೆ=6.. [ ]

Date of interview: ಸಂದರ್ಶನ ದಿನಾಂಕ: DAY ದಿನ [ ][ ] MONTH ತಿಂಗಳು [ ][ ] YEAR ವರ್ಷ [ ][ ][ ][ ]

Name and code of interviewer:______________________________ [ ][ ]
ಸಂದರ್ಶಕರ ಹೆಸರು ಮತ್ತು ಕೊಡ್

CONSENT FOR INTERVIEW:

YES/ಹೌದು.........1 ⟶ PROCEED WITH INTERVIEW/ಸಂದರ್ಶನ ಪ್ರಕ್ರಿಯೇ ನಡೆಸಿ
NO/ಇಲ್ಲಾ.........0 ⟶ STOP/ನಿಲ್ಲಿಸಿ

INTERVIEW START TIME (IN 24 HOURS SYSTEM): [ ][ ][ ][ ]
ಸಂದರ್ಶನ ಪ್ರಾರಂಬಗೊಂಡ ಸಮಯ (24 ಗಂಟೆಗಳಲ್ಲಿ)

INTERVIEW COMPLETE TIME (IN 24 HOURS SYSTEM): [ ][ ][ ][ ]
ಸಂದರ್ಶನ ಮುಖ್ತಾಯಗೊಂಡ ಸಮಯ (24 ಗಂಟೆಗಳಲ್ಲಿ)

SECTION II: EDITING AND DATA ENTRY
ವಿಭಾಗ ೨: ಪರಿಷ್ಕರಣೆ ಮತ್ತು ದತ್ತಾಂಶ ನಮೂದು

Name and code of Supervisor: ______________________________ [ ]
ಮೆಲ್ವೀಚಾರಕರ ಹೆಸರು ಮತ್ತು ಕೊಡ್

Date of scrutinizing the questionnaire: ಸಂದರ್ಶನ ದಿನಾಂಕ: DAY ದಿನ [ ][ ] MONTH ತಿಂಗಳು [ ][ ] YEAR ವರ್ಷ [ ][ ][ ][ ]

Signature of Supervisor:______________________________
ಮೆಲ್ವೀಚಾರಕರ ಸಹಿ

Name and code of Data entry person: ______________________________ [ ]
ದತ್ತಾಂಶ ನಮೂದಕರ ಹೆಸರು ಮತ್ತು ಕೊಡ್

Date of data entry: ದತ್ತಾಂಶ ನಮೂದಿಸಿದ ದಿನಾಂಕ: DAY ದಿನ [ ][ ] MONTH ತಿಂಗಳು [ ][ ] YEAR ವರ್ಷ [ ][ ][ ][ ]

Signature of data entry person:______________________________
ದತ್ತಾಂಶ ನಮೂದಕರ ಸಹಿ

SECTION III. DEMOGRAPHIC CHARACTERISTICS

ವಿಭಾಗ ೩: ಜನಸಂಖ್ಯೆಶಾಸ್ತ್ರೀಯ ಗುಣಲಕ್ಷಣಗಳು

NO.	QUESTIONS	CODING CATEGORIES	SKIP
301	How old are you now?<br>ಈಗ ನಿಮ್ಮ ವಯಸ್ಸೆಷ್ಟು?	AGE IN COMPLETED YEARS.. ☐☐<br>ಪೂರ್ಣಗೊಂಡ ವಯಸ್ಸು<br>DON'T KNOW/ಗೊತ್ತಿಲ್ಲ........... 98	
302	What is your religion?<br>ನಿಮ್ಮ ಧರ್ಮ ಯಾವುದು?	HINDU/ಹಿಂದೂ.................. 1<br>MUSLIM/ಮುಸ್ಲಿಂ .... 2<br>CHRISTIAN/ಕ್ರೀಶ್ಚಿಯನ್ .... 3<br>BUDDHIST/ಬೌದ್ದದರ್ಮ............ 4<br>JAIN/ಜೈನದರ್ಮ .... 5<br>OTHER/ಇತರೆ ___________________97<br>(SPECIFY<br>DON'T KNOW/ ಗೊತ್ತಿಲ .... 98<br>NO ANSWER/ ಪ್ರತಿಕ್ರೀಯೆ ಇಲ್ಲಾ 99	
303	Can you read and write?<br>ನಿಮಗೆ ಓದಲು ಬರೆಯಲು ಬರುತ್ತದೆಯೇ?	YES/ಹೌದು .... 1<br>NO/ಇಲ್ಲಾ 0	→305
304	What is the highest grade you have completed until now?<br>ಇದುವರೆಗೂ ನೀವು ಪಡೆದ ಉನ್ನತ ಶಿಕ್ಷಣದ ಹಂತ ಯಾವುದು?	GRADE/ತರಗತಿ............. ☐☐<br>INFORMAL EDUCATION..........00<br>ಅನೌಪಚಾರಿಕ ಶಿಕ್ಷಣ<br>DON'T KNOW/ ಗೊತ್ತಿಲ್ಲ .... 98<br>NO ANSWER/ ಪ್ರತಿಕ್ರಿಯೆ ಇಲ್ಲಾ....... 99	
305	What is your main source of income?<br>ನಿಮ್ಮ ಆದಾಯದ ಪ್ರಮುಖ ಮೂಲ ಯಾವುದು?	UNEMPLOYED/ನಿರುದ್ಯೋಗಿ.......... 1<br>STUDENT/ವಿಧ್ಯಾರ್ಥಿ .... 2<br>SELF EMPLOYED PROFESSSIONAL. 3<br>ಸ್ವಂತ ಉದ್ಯೋಗಿ<br>NON-AGRICULTURAL LABOUR..... 4<br>ಕೃಷಿಯೇತರ ಕಾರ್ಮಿಕ<br>BUSINESS/TRADE/ವ್ಯಾಪಾರ .... 5<br>SERVICE (GOVT/PVT) .... 6<br>ಸರ್ಕಾರಿ/ಖಾಸಗಿ ಸೇವೆ<br>MASSAGER (MASSEUR)/ಮಸಾಜರ್... 7<br>PUN(SEX WORK) /ಲೈಂಗಿಕ ವೃತ್ತಿ .... 8<br>TRANSPORT WORKER/ಸಾರಿಗೆ ನೌಕರ... 9<br>BASTI/ಬಸ್ತಿ .... 10<br>OTHER/ಇತರೆ____________________97<br>NO ANSWER/ ಪ್ರತಿಕ್ರೀಯೆ ಇಲ್ಲಾ 99	
306	How do you identify yourself?<br>ನಿಮ್ಮನ್ನು ನೀವು ಹೇಗೆ ಗುರುತಿಸಿಕೊಳ್ಳುತ್ತೀರಿ?<br><br>READ RESPONSE CATEGORIES AND MARK ONLY ONE CODE<br>ಪ್ರತಿಕ್ರೀಯೇಯನ್ನ ಓದಿ ಮತ್ತು ಒಂದನ್ನು ಮಾತ್ರ ದಾಖಲಿಸಿ	HIJRA NIRVAN/ನಿರ್ವಾಣ ಹಿಜಿಡ .... 1<br>HIJRA AKWA/ಅಕ್ವ ಹಿಜಿಡ .... 2<br>KOTHI/ಕೊಥಿ .... 3<br>OTHERS/ಇತರೆ................. 97<br>(SPECIFY ANSWER)<br>NO ANSWER/ ಪ್ರತಿಕ್ರೀಯೇ ಇಲ್ಲಾ 99	
307	Have you ever been married to a woman?<br>ನಿವು ಯಾವಾಗಲಾದರು ಮಹಿಳೆಯನ್ನು ಮದುವೆಯಾಗಿದ್ದೀರಾ?	YES/ಹೌದು .... 1<br>NO/ಇಲ್ಲಾ 0	→ 310
308	Whose decision was it for you to get married?<br>ನೀವು ಮದುವೆಯಾಗಬೇಕೆಂದು ನಿರ್ಧರಿಸಿದವರು ಯಾರು?	FAMILY/ಕುಟುಂಬ................ 1<br>MYSELF/ಸ್ವತ: ನಾನೇ.............. 2<br>OTHERS/ಇತರೆ__________________ 97	

NO.	QUESTIONS	CODING CATEGORIES	SKIP
309	What is your current marital status?<br>ನಿಮ್ಮ ಸಧ್ಯದ ವೈವಾಹಿಕ ಸ್ಥಿತಿ ಏನು?	MARRIED-LIVING WITH SPOUSE .. 1<br>ಮದುವೆ ಆಗಿದೆ- ಸಂಗಾತಿಯೊಂದಿಗೆ ವಾಸವಾಗಿದ್ದೇನೆ<br>MARRIED-LIVING WITH PARTNER OTHER THAN SPOUSE .... 2<br>ಮದುವೆ ಆಗಿದೆ- ಸಂಗಾತಿಯೊಂದಿಗೆ ವಾಸವಾಗಿದ್ದೇನೆ ಆದರೆ ವಿವಾಹಿತ ಸಂಗಾತಿಯಲ್ಲ<br>MARRIED-LIVING ALONE .... 3<br>ಮದುವೆಯಾಗಿದೆ ಆದರೆ ಒಂಟಿಯಾಗಿದ್ದೇನೆ<br>DIVORCED-LIVING ALONE ...... 4<br>ವಿವಾಹ ವಿಚ್ಛೇದನವಾಗಿದೆ-ಒಂಟಿಯಾಗಿದ್ದೇನೆ<br>DIVORCED-LIVING WITH OTHER PARTNER .... 5<br>ವಿವಾಹ ವಿಚ್ಛೇದನವಾಗಿದೆ-ಬೇರೆ ಸಂಗಾತಿಯೊಂದಿಗೆ ವಾಸವಾಗಿದ್ದೇನೆ<br>WIDOWED-LIVING ALONE .... 6<br>ವಿದವೆ/ವಿದುರ- ಒಂಟಿಯಾಗಿದ್ದೇನೆ<br>WIDOWED-LIVING WITH PARTNER . 7<br>ವಿದವೆ/ವಿದುರ-ಸಂಗಾತಿಯೊಂದಿಗೆ ವಾಸವಾಗಿದ್ದೇನೆ<br>OTHER/ಇತರೆ__________________ 97<br>NO ANSWER/ ಪ್ರತಿಕ್ರಿಯೆ ಇಲ್ಲಾ....... 99	1–3 → 311
310	Do you intend to get married to a woman in the future?<br>ನೀವು ಮುಂದೆ ಮಹಿಳೆಯನ್ನು ಮದುವೆಯಾಗಲು ಬಯಸುವಿರಾ?<br>**[DON'T ASK THIS QUESTION TO HIJRA]**<br>**[ಹಿಜಿಡ ಆಗಿದ್ದರೆ ಈ ಪ್ರಶ್ನೆ ಕೀಳಬೇಡಿ]**	YES/ಹೌದು .... 1<br>NO/ಇಲ್ಲಾ .... 0<br>CAN'T SAY/ಹೇಳಲಾರೆ 2	
311	What is your current living status?<br>ನೀವು ಈಗ ಯಾರ ಜೊತೆ ವಾಸಿಸುತ್ತಿದ್ದೀರಾ?	LIVING ALONE/ .... 1<br>ಒಬ್ಬಂಟಿಯಾಗಿ ಜೀವಿಸುತ್ತಿರುವೆ<br>LIVING WITH FAMILY .... 2<br>ಕುಟುಂಬದೊಂದಿಗೆ ಜೀವಿಸುತ್ತಿರುವೆ<br>LIVING WITH SPOUSE .... 3<br>ಸಂಗಾತಿಯೊಂದಿಗೆ ಜೀವಿಸುತ್ತಿರುವೆ<br>LIVING WITH MALE SEXUAL PARTNER .... 4<br>ಪುರುಷ ಲೈಂಗಿಕ ಸಂಗಾತಿಯೊಂದಿಗೆ ಜೀವಿಸುತ್ತಿರುವೆ<br>LIVING WITH MALE SEXUAL PARTNER AND SPOUSE TOGETHER..5<br>ಪುರುಷ ಲೈಂಗಿಕ ಸಂಗಾತಿ ಮತ್ತು ಗಂಡ/ಹೆಂಡತಿಯೊಂದಿಗೆ ಜೀವಿಸುತ್ತಿರುವೆ<br>LIVING WITH FRIENDS .... 6<br>ಸ್ನೇಹಿತನೊಂದಿಗೆ ಜೀವಿಸುತ್ತಿರುವೆ<br>LIVING WITH GURU/ .... 7<br>ಗುರುವಿನೊಂದಿಗೆ ವಾಸ<br>OTHER/ಇತರೆ __________________97	
312	Do you currently live here in Bangalore?<br>ನೀವು ಈಗ ಸದ್ಯ ಬೆಂಗಳೂರಿನಲ್ಲಿ ವಾಸವಾಗಿರುವಿರಾ?	YES/ಹೌದು .... 1<br>NO/ಇಲ್ಲಾ 0	0 → 314
313	How long have you been living in this place?<br>ಈ ಸ್ಥಳದಲ್ಲಿ ನೀವು ಎಷ್ಟು ಕಾಲದಿಂದ ವಾಸವಾಗಿರುವಿರಿ?	LESS THAN A YEAR .... 00<br>ಒಂದು ವರ್ಷಕ್ಕಿಂತ ಕಡಿಮೆ ಅವಧಿಯಿಂದ<br>NUMBER OF YEARS .... [ ][ ]<br>ವರ್ಷಗಳ ಸಂಖ್ಯೆ<br>SINCE BIRTH/ಹುಟ್ಟಿದಾಗಿನಿಂದ...... 97<br>DON'T KNOW/ ಗೊತ್ತಿಲ್ಲಾ 98	97, 98 → 401

NO.	QUESTIONS	CODING CATEGORIES	SKIP
314	What was the main reason for your move to Bangalore?<br>ನೀವು ಬೆಂಗಳೂರಿಗೆ ವಲಸೆ ಬರಲು ಕಾರಾಣವಾದ ಮುಖ್ಯ ಅಂಶ ಯಾವುದು?	RELATED TO WORK .... 1<br>ಕೆಲಸ ಸಂಬಂಧ<br>RELATED TO FAMILY .... 2<br>ಕುಟುಂಬ ಸಂಬಂಧ<br>RELATED TO COMMUNITY .... 3<br>ಸಮುದಾಯ ಸಂಬಂಧ<br>RELATED TO NEIGHBOURHOOD ... 4<br>ನೆರೆಹೊರೆಯವರ ಸಂಬಂಧ<br>OTHER/ಇತರೆ__________________97<br>(SPECIFY)<br>DON'T KNOW/ ಗೊತ್ತಿಲ .... 98<br>NO ANSWER/ ಪ್ರತಿಕ್ರೀಯೆ ಇಲ್ಲಾ 99	

SECTION IV: BODY IMAGE
ವಿಭಾಗ ೪: ದೇಹದ ಪರಿಕಲ್ಪನೆ

Now I would like to ask some question on your perception about your body image.
ನಿಮ್ಮ ದೇಹದ ಪರಿಕಲ್ಪನೆಯ ಕುರಿತಾದ ನಿಮ್ಮ ಮನೋಭಾವನೆಯನ್ನು ತಿಳಿಯಲು ಕೆಲವು ಪ್ರಶ್ನೆಗಳನ್ನ ಕೇಳುತ್ತೇನೆ.

NO.	QUESTIONS	CODING CATEGORIES	SKIP
401	Do you feel happy with your current physical appearance?<br>ನಿಮಗೆ ನಿಮ್ಮ ಸಧ್ಯದ ರೂಪದ ಬಗ್ಗೆ ಸಂತೃಪ್ತಿಯಿದೆಯೇ?	VERY MUCH/ಅತಿ ಹೆಚ್ಚು .... 1<br>SOMEWHAT/ಸ್ವಲ್ಪಮಟ್ಟಿಗೆ............ 2<br>VERY LITTLE/ತುಂಬಾ ಕಡಿಮೆ .... 3<br>NOT AT ALL/ಇಲ್ಲವೆ ಇಲ್ಲಾ......... 4<br>INDIFFERENT/ಪರವಾಗಿಲ್ಲ 5	
402	Do you feel that people appreciate your physical appearance?<br>ನಿಮ್ಮ ದೈಹಿಕ ರೂಪವನ್ನು ಜನ ಹೊಗಳುತ್ತಾರೆಂದು ನಿಮಗೆ ಅನ್ನಿಸುತ್ತದೆಯೇ?	YES/ಹೌದು .... 1<br>NO/ಇಲ್ಲಾ .... 0<br>DON'T KNOW/CAN'T SAY .... 8<br>ಗೊತ್ತಿಲ್ಲಾ/ಹೇಳಲಾರೆ<br>NO ANSWER/ ಪ್ರತಿಕ್ರೀಯೇ ಇಲ್ಲಾ 99	
403	Is there anything you would like to change about your physical appearance?<br><br>ನಿಮ್ಮ ದೈಹಿಕ ರೂಪದಲ್ಲಿ ಏನನ್ನಾದರು ಬದಲಾಯಿಸಿಕೊಳ್ಳಲು ಇಚ್ಛಿಸುವಿರಾ?<br><br>**MULTIPLE OPTIONS ARE POSSIBLE**<br>**ಒಂದಕ್ಕಿಂತ ಹೆಚ್ಚು ಪ್ರತಿಕ್ರೀಯೆಗಳ ಸಾಧ್ಯತೆಯಿದೆ**	BREASTS/ಸ್ಥನ/ಮೊಲೆ .... A<br>BODY HAIR REMOVAL<br>ದೇಹದ ಕೊದಲು ತೆಗೆಯುವುದು .... B<br>NIRVAN/ನಿರ್ವಾಣಾ .... C<br>VAGINA/ಯೋನಿ .... D<br>VOICE CHANGE/ ಧ್ವನಿ ಬದಲಾವಣೆ ... E<br>SILICON IMPLANTS<br>(HIPS BUTTOCKS)/ಪಿರ್ರೆಗಳ ಕಸಿ..... F<br>NO CHANGES REQUIRED......... G<br>ಯಾವ ಬದಲಾವಣೆಯು ಅವಶ್ಯವಿಲ್ಲಾ<br>OTHER/ಇತರೆ____________________X	 <br><br><br><br><br><br><br>→ 406
404	Do you have support from any person for the changes required in your physical appearance?<br>ನಿಮ್ಮ ದೈಹಿಕ ರೂಪದಲ್ಲಿ ಅವಶ್ಯವಿರುವ ಬದಲಾವಣೆಗೆ ನಿಮಗೆ ಯಾರಿಂದಲಾದರು ಬೆಂಬಲವಿದೆಯೇ?<br><br>**MULTIPLE OPTIONS ARE POSSIBLE**<br>**ಒಂದಕ್ಕಿಂತ ಹೆಚ್ಚು ಪ್ರತಿಕ್ರೀಯೆಗಳ ಸಾಧ್ಯತೆಯಿದೆ**	FAMILY/ಕುಟುಂಬ................ A<br>FRIENDS/ಸ್ನೆಹಿತರು.............. B<br>NEIGHBOURS/ನೆರೆಹೊರೆಯವರು ...... C<br>GURU/ಗುರು.................... D<br>PARTNER/ಸಂಗಾತಿ .... E<br>OTHERS/ಇತರೆ __________________X<br>NO SUPPORT/ಬೆಂಬಲವಿಲ್ಲಾ .... Y<br>NO ANSWER/ ಪ್ರತಿಕ್ರೀಯೇ ಇಲ್ಲಾ 99	
405	Who will financially support you for changes in your physical appearance?<br>ನಿಮ್ಮ ದೈಹಿಕ ರೂಪದ ಬದಲಾವಣೆಗೆ ನಿಮಗೆ ಯಾರು ಹಣಕಾಸಿನ ಸಹಾಯ ಮಾಡುತ್ತಾರೆ?<br>**MULTIPLE OPTIONS ARE POSSIBLE**<br>**ಒಂದಕ್ಕಿಂತ ಹೆಚ್ಚು ಪ್ರತಿಕ್ರೀಯೆಗಳ ಸಾಧ್ಯತೆಯಿದೆ**	SELF/ಸ್ವಂತ .... A<br>GURU/ಗುರು.................... B<br>PARTNER/ಸಂಗಾತಿ .... C<br>OTHERS/ಇತರೆ ________________X<br>NO SUPPORT/ಬೆಂಬಲವಿಲ್ಲಾ .... Y<br>NO ANSWER/ ಪ್ರತಿಕ್ರೀಯೇ ಇಲ್ಲಾ 99	
406	Have you done any changes in your physical appearance before? ನೀವು ಈ ಮೊದಲು ನಿಮ್ಮ ದೈಹಿಕ ರೂಪದಲ್ಲಿ ಏನಾದರು ಬದಲಾವಣೆ ಮಾಡಿಕೊಂಡಿರುವಿರಾ?	YES/ಹೌದು .... 1<br>NO/ಇಲ್ಲಾ 0	 → 501

NO.	QUESTIONS	CODING CATEGORIES	SKIP
407	How do you feel about the physical changes you did before?<br>ಈ ಮೊದಲು ನಿಮ್ಮ ದೈಹಿಕ ರೂಪದಲ್ಲಿ ಬದಲಾವಣೆ ಮಾಡಿಕೊಂಡಿರುವುದರ ಬಗ್ಗೆ ನಿಮ್ಮ ಭಾವನೆ ಏನು?<br>PROBE THE ANSWER/ಉತ್ತರವನ್ನ ಆಳವಾಗಿ ಶೋಧಿಸಿ	FELT GOOD/ಉತ್ತಮ ಭಾವನೆ .... 1<br>DID NOT FEEL GOOD .... 2<br>ಒಳ್ಳೆ ಭಾವನೆ ಇಲ್ಲಾ<br>INDIFFERENT/ಪರವಾಗಿಲ್ಲ .... 3<br>OTHER/ಇತರೆ ___________________97	

SECTION V: SELF ESTEEM
ವಿಭಾಗ ೫: ಸ್ವ-ಗೌರವ

In this section I would like to ask some questions on your physical appearance, sexuality, family relation, and social life.
ಈ ವಿಭಾಗದಲ್ಲಿ ನಾನು ನಿಮ್ಮ ದೈಹಿಕ ರೂಪ, ಲೈಂಗಿಕತೆ, ಕುಟುಂಬದೊಂದಿಗಿನ ಸಂಬಂದ ಮತ್ತು ಸಾಮಾಜಿಕ ಜೀವನದ ಬಗ್ಗೆ ಕೆಲವು ಪ್ರಶ್ನೆಗಳನ್ನ ಕೇಳಲು ಇಚ್ಚಿಸುತ್ತೇನೆ.

NO.	QUESTIONS	CODING CATEGORIES				SKIP
	**A. SEXUALITY ASPECTS**<br>**ಎ. ಲೈಂಗಿಕತೆಯ ಅಂಶಗಳು**	**NEVER**<br>ಯಾವಾಗಲು ಇಲ್ಲಾ	**SOME-TIMES**<br>ಕೆಲವು ಸಮಯ	**ALWAYS**<br>ಯಾವಾ-ಗಲು ಇದೆ	**NA**<br>ಪ್ರತಿಕ್ರೀಯೇ ಇಲ್ಲಾ	
501	Do you feel that people will not like to meet you if they find out about your sexual identity<br>ನಿಮ್ಮ ಲೈಂಗಿಕತೆಯ ಬಗ್ಗೆ ಜನಗಳಿಗೆ ತಿಳಿದರೆ ಅವರು ನಿಮ್ಮನ್ನು ಭೇಟಿಯಾಗಲು ಇಷ್ಟಪಡುವುದಿಲ್ಲಾ ಎಂದು ನಿಮಗನ್ನಿಸುತ್ತದೆಯೇ.	0	1	2	9	
502	Are you not confident of yourself as a sexual partner<br>ಲೈಂಗಿಕ ಸಂಗಾತಿಯಾಗಿ ನಿಮ್ಮ ಬಗ್ಗೆ ನಿಮಗೆ ವಿಸ್ವಾಸವಿಲ್ಲಾವೆ	0	1	2	9	
503	Do you feel unhappy about your sexual relationship<br>ನಿಮ್ಮ ಲೈಂಗಿಕ ಸಂಬಂಧದ ಬಗ್ಗೆ ನಿಮಗೆ ದುಖಃವಿದೆಯೇ	0	1	2	9	
504	Do you feel unsatisfied with your sex life<br>ನಿಮ್ಮ ಲೈಂಗಿಕ ಜೀವನವು ನಿಮಗೆ ಅತೃಪ್ತಿ ಎಂದೆನ್ನಿಸಿದೆಯೇ.	0	1	2	9	
	**B. FAMILY**<br>**ಬಿ. ಕುಟುಂಬ**					
505	Do you hide your sexual identity from your family members<br>ನಿವು ನಿಮ್ಮ ಲೈಂಗಿಕ ಗುರುತಿಸುವಿಕೆಯನ್ನು ನಿಮ್ಮ ಕುಟುಂಬದಿಂದ ಮುಚ್ಚಿಟ್ಟಿರುವಿರಾ.	0	1	2	9	
506	Do you feel that you are not needed in your family<br>ನೀವು ನಿಮ್ಮ ಕುಟುಂಬಕ್ಕೆ ಬೇಕಾಗಿಲ್ಲಾ ಎಂದು ನಿಮಗನ್ನಿಸಿದೆಯೇ.	0	1	2	9	
507	Do you feel disturbed when your family members do not approve of your identity<br>ನಿಮ್ಮ ಕುಟುಂಬದವರು ನಿಮ್ಮ ಲೈಂಗಿಕ ಗುರುತಿಸುವಿಕೆಯನ್ನ ಒಪ್ಪಿಲ್ಲವೆಂದಾಗ ಗೊಂದಲಗೊಂಡಿರುವಿರಾ.	0	1	2	9	
508	Are you happy that your parents care about you<br>ನಿಮ್ಮ ಪೋಷಕರು ನಿಮ್ಮ ಬಗ್ಗೆ ಕಾಳಜಿ ವಹಿಸುವುದು ನಿಮಗೆ ಸಂತೋಷ ತಂದಿದೆಯೇ.	0	1	2	9	

NO.	QUESTIONS	CODING CATEGORIES				SKIP
	**C. SOCIAL ASPECTS**<br>**ಸಿ. ಸಾಮಾಜಿಕ ಅಂಶಗಳು**	**NEVER**<br>ಯಾವಾಗಲು ಇಲ್ಲಾ	**SOME-TIMES**<br>ಕೆಲವು ಸಮಯ	**ALWAYS**<br>ಯಾವಾ-ಗಲು ಇದೆ	**NA**<br>ಪ್ರತಿಕ್ರೀಯೇ ಇಲ್ಲಾ	
509	Do you feel that you can not make friends in the society<br>ಸಮಾಜದಲ್ಲಿ ಸ್ನೆಹಿತರನ್ನು ಪಡೆಯಲು ಸಾದ್ಯವಿಲ್ಲ ಎಂದು ನಿಮಗನ್ನಿಸಿದೆಯ.	0	1	2	9	
510	Do you feel uncomfortable when you are with people outside your community<br>ನಿಮ್ಮ ಸಮುದಾಯದವರನ್ನು ಬಿಟ್ಟು ಬೇರೆಯವರೊಂದಿಗೆ ಇರುವುದು ನಿಮಗೆ ಹಿತವಲ್ಲ ಎಂದೆನ್ನಿಸಿದೆಯಾ.	0	1	2	9	
511	Do you feel out of place when you go to attend social functions<br>ಸಾಮಾಜಿಕ ಕಾರ್ಯಕ್ರಮಗಳಲ್ಲಿ ಭಾಗವಹಿಸಲು ಹೋಗುವುದು ನಿಮಗೆ ಬೇರೆ ಸ್ಥಳಗಳಿಗೆ ಹೋದಂತೆ ಎಂದೆನ್ನಿಸುತ್ತದೆಯೇ.	0	1	2	9	
512	Are you afraid of being rejected by your community<br>ನಿಮ್ಮನ್ನು ನಿಮ್ಮ ಸಮುದಾಯವು ತಿರಸ್ಕರಿಸುವ ಭಯವಿದೆಯೇ.	0	1	2	9	
513	Are you confident of facing the police<br>ನೀವು ಪೋಲಿಸರನ್ನು ಏದುರಿಸುವ ವಿಶ್ವಾಸಹೊಂದಿರುವಿರಾ.	0	1	2	9	
514	Do you feel that you are fairly treated at public facilities (like hospitals, bus, shopping complexes)<br>ಸಾಮಾಜಿಕ ಸೌಲತ್ತುಗಳಿಗೆ ಸಂಭಂಧಿಸಿದಂತೆ ನಿಮ್ಮನ್ನು ಸಮಾನವಾಗಿ ನಡೆಸಿಕೊಳ್ಳಲಾಗುವುದು ಎಂಬ ವಿಶ್ವಾಸ ನಿಮಗಿದೆಯೇ (ಆಸ್ಪತ್ರೆ, ಬಸ್, ಶಾಪಿಂಗ್ ಕಾಂಪ್ಲೆಕ್ಸ್)	0	1	2	9	
515	Do you wish you could be treated like other people in the society<br>ನಿಮ್ಮನ್ನು ಸಮಾಜದಲ್ಲಿನ ಇತರರಂತೆ ನಡೆಸಿಕೊಳ್ಳಬೇಕು ಎಂದು ಆಶಿಸುತ್ತೀರಾ.	0	1	2	9	
	**D. SELF SCALE**<br>**ಡಿ. ಸ್ವಯಂ ಮೌಲ್ಯಮಾಪನ**					
516	Do you feel that you are not a useful person<br>ನೀವೊಬ್ಬ ಉಪಯುಕ್ತ ವ್ಯಕ್ತಿ ಅಲ್ಲ ಎಂದು ನಿಮಗನ್ನಿಸುತ್ತದೆಯೇ	0	1	2	9	
517	Do you feel satisfied with your ability to stand up for your rights<br>ನಿಮ್ಮ ಹಕ್ಕುಗಳ ಬಗ್ಗೆ ಹೊರಾಡುವ ನಿಮ್ಮ ಶಕ್ತಿಯ ಕುರಿತು ನಿಮಗೆ ಸಮಾದಾನವಿದೆಯಾ.	0	1	2	9	
518	Do you feel useless when you cannot perform well in difficult situations<br>ಕಷ್ಟದ ಸನ್ನಿವೇಶದಲ್ಲಿ ಸರಿಯಾಗಿ ಕಾರ್ಯನಿರ್ವಹಿಸದಿದ್ದಾಗ ನೀವು ಅನುಪಯುಕ್ತ ಎಂದೆನ್ನಿಸುತ್ತೀರಾ.	0	1	2	9	
519	Do you like and accept yourself right now, the way you are<br>ನೀವು ನಿಮ್ಮ ಈಗಿನ ಸ್ಥಿತಿಯನ್ನು ಇಷ್ಟಪಡುತ್ತೀರಾ ಮತ್ತು ಸ್ವೀಕರಿಸುತ್ತೀರಾ.	0	1	2	9	
520	Is it very important to you to feel independent<br>ನೀವು ಸ್ವತಂತ್ರವಾಗಿರಬೇಕು ಎಂಬುದು ನಿಮಗೆ ಅತ್ಯಂತ ಮಹತ್ವವಾದುದೆ.	0	1	2	9	

SECTION VI: RELATIONSHIPS
ವಿಭಾಗ ೬: ಸಂಬಂಧ

Now I would like to ask some questions on your relationship with your partner and other community members
ನಿಮ್ಮ ಸಂಗಾತಿ ಮತ್ತು ಇತರ ಸಮುದಾಯ ಸದಸ್ಯರೊಂದಿಗಿನ ನಿಮ್ಮ ಸಂಬಂಧದ ಕುರಿತು ಕೆಲವು ಪ್ರಶ್ನೆಗಳನ್ನ ನಾನು ಈ ವಿಭಾಗದಲ್ಲಿ ಕೇಳಲು ಇಚ್ಛುಸುತ್ತೇನೆ.

NO.	QUESTIONS	CODING CATEGORIES				SKIP
		NEVER<br>ಯಾವಾಗಲು ಇಲ್ಲಾ	SOME-TIMES<br>ಕೆಲವು ಸಮಯ	ALWAYS<br>ಯಾವಾ-ಗಲು ಇದೆ	NA<br>ಪ್ರತಿಕ್ರೀಯೇ ಇಲ್ಲಾ	
601	Do you find it easy to get emotionally close to others<br>ಇತರರೋಂದಿಗೆ ಭಾವನಾತ್ಮಕವಾಗಿ ಹತ್ತಿರವಾಗುವುದು ನಿಮಗೆ ಸುಲಭವೆಂದೆನ್ನಿಸುತ್ತದೆಯೇ.	0	1	2	9	
602	Do you worry that you will be hurt if you allow yourself to become too close to others<br>ಇತರರಿಗೆ ನೀವು ಅತ್ಯಂತ ಹತ್ತಿರವಾದರೆ ನಿಮಗೆ ನೋವಾಗುತ್ತದೆ ಎಂದೆನ್ನಿಸುತ್ತದೆಯೇ.	0	1	2	9	
603	Do you worry about being alone<br>ಒಂಟಿಯಾಗಿರುವುದು ನಿಮಗೆ ನೋವಾಗುತ್ತದೆಯೇ.	0	1	2	9	
604	Do you worry that you panthi/partner doesn’t really love you<br>ನಿಮ್ಮ ಸಂಗಾತಿ ನಿಮ್ಮನ್ನು ನಿಜವಾಗಿಯು ಪ್ರೀತಿಸುವುದಿಲ್ಲ ಎಂಬುದು ನಿಮ್ಮನ್ನು ಕಾಡುತ್ತಿದೆಯೇ	0	1	2	9	
605	Do you find difficult to balance your married life and your relationships with men<br>ನಿಮ್ಮ ವೈವಾಹಿಕ ಸಂಬಂಧ ಮತ್ತು ಬೇರೆ ಪುರುಷರೊಂದಿಗಿನ ನಿಮ್ಮ ಸಂಬಂಧವನ್ನು ಸರಿದೂಗಿಸುವುದು ನಿಮಗೆ ಕಷ್ಟವೆಂದೆನ್ನಿಸುತ್ತದೆಯೇ.	0	1	2	9	

SECTION VII: ALCOHOL CONSUMPTION
ವಿಭಾಗ ೭: ಮಧ್ಯಸೇವನೆ

NO.	QUESTIONS	CODING CATEGORIES	SKIP
701	Do you consume alcohol?<br>ನೀವು ಮಧ್ಯಸೇವನೆ ಮಾಡುತ್ತೀರ?	NEVER CONSUMED ALCOHOL ...... 1<br>ಯಾವಾಗಲು ಮಧ್ಯ ಸೇವಿಸಲಿಲ್ಲಾ<br>EVERYDAY/ಪ್ರತಿದಿನ .... 2<br>AT LEAST ONCE A WEEK ...... 3<br>ಕನಿಷ್ಟ ವಾರಕ್ಕೆ ಒಮ್ಮೆ<br>LESS THAN ONCE A WEEK ...... 4<br>ಒಂದು ವಾರಕ್ಕಿಂತ ಕಡಿಮೆ ಅವಧಿಗೆ<br>NO ANSWER/ಪ್ರತಿಕ್ರೀಯೇ ಇಲ್ಲಾ 99	→ 801
702	With whom do you normally drink with?<br>ನೀವು ಸಾಮಾನ್ಯವಾಗಿ ಯಾರೊಂದಿಗೆ ಮಧ್ಯಸೇವನೆ ಮಾಡುತ್ತೀರಿ?<br><br>**MULTIPLE RESPONSES ARE POSSIBLE**<br>**ಒಂದಕ್ಕಿಂತ ಹೆಚ್ಚು ಪ್ರತಿಕ್ರೀಯೇಗಳು ಸಾಧ್ಯವಿದೆ**	MALE PARTNER/ಪುರುಷ ಸಂಗಾತಿ .... A<br>FRIENDS/ಸ್ನೇಹಿತರು .... B<br>CLIENTS/ಗಿರಾಕಿಗಳು .... C<br>GURU/ಗುರು .... D<br>OTHER/ ಇತರೆ____________________97<br>(SPECIFY)<br>NO ANSWER/ಪ್ರತಿಕ್ರೀಯೇ ಇಲ್ಲಾ 99	
703	Why do you consume alcohol?<br><br>ನೀವು ಯಾಕೆ ಮಧ್ಯಸೇವನೆ ಮಾಡುತ್ತೀರಿ?<br><br>MULTIPLE RESPONSES ARE POSSIBLE<br>ಒಂದಕ್ಕಿಂತ ಹೆಚ್ಚು ಪ್ರತಿಕ್ರೀಯೇಗಳು ಸಾಧ್ಯವಿದೆ	TO CELEBRATE HAPPINESS ..... A<br>ಸಂತೋಷವನ್ನ ಆಚರಿಸಲು<br>WHEN I AM SAD/ನನಗೆ ಬೇಸರವಾದಾಗ .. B<br>BEFORE HAVING SEX .... C<br>ಲೈಂಗಿಕ ಸಂಪರ್ಕ ಮಾಡುವ ಮೊದಲು<br>TO ACCOMPANY OTHERS .... D<br>ಇತರರಿಗೆ ಕಂಪನಿಕೊಡಲು<br>OTHER/ ಇತರೆ____________________97<br>NO ANSWER/ಪ್ರತಿಕ್ರೀಯೇ ಇಲ್ಲಾ 99	

NO.	QUESTIONS	CODING CATEGORIES	SKIP
704	Do you panic when you do not have a drink when you need it?<br>ನಿಮಗೆ ಕುಡಿಯಬೇಕೆಂದಾಗ ಮಧ್ಯಸೇವನೆ ಮಾಡದಿದ್ದರೆ ನಿಮ್ಮ ಮೈ-ಕೈ ನಡುಕವಾಗುತ್ತದೆಯೇ?	YES/ಹೌದು .... 1<br>NO/ಇಲ್ಲಾ .... 0<br>IT NEVER HAPPENED .... 8<br>ಯಾವಾಗಲು ಹಾಗೆ ಆಗಿಲ್ಲಾ	
705	Have you experienced blackouts or loss of memory due to drinking in past six months?<br>ಕಳೆದ ಆರು ತಿಂಗಳಿನಲ್ಲಿ ಮಧ್ಯಸೇವನೆಯಿಂದ ನಿಮಗೆ ನೆನಪಿನ ಶಕ್ತಿಕಳೆದುಕೊಂಡ ಅನುಭವವಾಗಿದೆಯೇ?	YES/ಹೌದು .... 1<br>NO/ಇಲ್ಲಾ 0	

SECTION VIII: HEALTH
ವಿಭಾಗ VIII: ಆರೋಗ್ಯ

Now I would like to discuss about your health status and the treatment seeking behaviour. ನಾನು ಈಗ ನಿಮ್ಮ ಆರೋಗ್ಯ ಪರಿಸ್ಥಿತಿ ಮತ್ತು ಚಿಕಿತ್ಸೆ ಪಡೆದುಕೊಳ್ಳುವ ನಡವಳಿಕೆಯ ಕುರಿತು ಚರ್ಚಿಸಲು ಇಚ್ಛಿಸುತ್ತೇನೆ.

NO.	QUESTIONS	CODING CATEGORIES	SKIP
801	Did you ever face any health related problems?<br>ನೀವು ಯಾವಾಗಲಾದರು ಆರೋಗ್ಯಕ್ಕೆ ಸಂಬಂಧಿಸಿದ ತೊಂದರೆ ಅನುಭವಿಸಿರುವಿರ?	NEVER/ ಯಾವಾಗಲು ಇಲ್ಲಾ.......... 0<br>YES, CURRENTLY SUFFERING.... 1<br>ಹೌದು, ಈಗ ಸಧ್ಯ ಅನುಭವಿಸುತ್ತಿದ್ದೇನೆ<br>WITHIN LAST 6 MONTHS .... 2<br>ಕಳೆದ ೬ ತಿಂಗಳಿನಿಂದ<br>WITHIN A YEAR/ ಒಂದು ವರ್ಷದಿಂದ.. 3<br>BEYOND 1 YEAR/ಒಂದು ವರ್ಷಕ್ಕಿಂತ ಹೆಚ್ಚು...4<br>CAN'T REMEMEMBER/ ನೆನಪಿಲ್ಲಾ 8	→804
802	What kind of health related problem you faced?<br>ಆರೊಗ್ಯ ಸಂಬಂಧಿಸಿದ ಯಾವ ತೊಂದರೆಗಳನ್ನ ಅನುಭವಿಸಿರುವಿರಿ?<br><br>**[MULTIPLE RESPONSES ARE POSSIBLE]**<br>**[ಒಂದಕ್ಕಿಂತ ಹೆಚ್ಚು ಪ್ರತಿಕ್ರೀಯೇಗಳು ಸಾಧ್ಯವಿದೆ]**	URETHRAL DISCHARGE .... A<br>ಜನನಾಂಗದಲ್ಲಿ ಸ್ರಾವ<br>GENITAL ULCERS/SORES ...... B<br>ಜನನಾಂಗದಲ್ಲಿ ಹುಣ್ಣು<br>SWELLING IN GROIN AREA ..... C<br>ತೊಡೆಸಂದಿಯಲ್ಲಿ ಬಾವು<br>BURNING PAIN ON URINATION . D<br>ಉರಿ ಮೂತ್ರ<br>CAN NOT RETRACT FORESKIN .. E<br>ಮುಂದೊಗಲನ್ನು ಸರಿಸಲು ಸಾಧ್ಯವಿಲ್ಲಾ<br>COUGH, COLD OR FEVER ...... F<br>ಕೆಮ್ಮು, ಶೀತ ಅಥವ ಜ್ವರ<br>HYPERTENSION/ ಉದ್ವೇಗ.......... G<br>DIABETES, BLOOD PRESSURE ... H<br>ಮಧುಮೇಹ, ರಕ್ತದೊತ್ತಡ<br>OTHER/ ಇತರೆ____________________97<br>NO ANSWER/ಪ್ರತಿಕ್ರೀಯೇ ಇಲ್ಲಾ 99	
803	What did you do **last time** when you had any health related problem?<br>**ಕಳೆದ ಬಾರಿ** ಯಾವುದೆ ಆರೋಗ್ಯ ಸಮಸ್ಯೆ ಉಂಟಾದಗ ನೀವು ಏನು ಮಾಡಿದಿರಿ?<br><br>**[MULTIPLE RESPONSES ARE POSSIBLE]**<br>**[ಒಂದಕ್ಕಿಂತ ಹೆಚ್ಚು ಪ್ರತಿಕ್ರೀಯೇಗಳು ಸಾಧ್ಯವಿದೆ]**	SOUGHT ADVICE/MEDICINE FROM CBO/NGO FACILITY/ ಸಿ.ಬಿ.ಒ/ಎನ್.ಜಿ.ಒ ದಿಂದ ಸಲಹೆ/ಔಷದಿ ಪಡೆದುಕೊಂಡೆ....... A<br>SOUGHT ADVICE/MEDICINE FROM GOVERNMENT FACILITY/ ಸರ್ಕಾರಿ ಸೌಲತ್ತಿನಿಂದ ಸಲಹೆ/ಔಷದಿ ಪಡೆದುಕೊಂಡೆ... B<br>SOUGHT ADVICE/MEDICINE FROM PRIVATE FACILITY ಖಾಸಗಿ ಸೌವಲತ್ತಿನಿಂದ ಸಲಹೆ/ಔಷದಿ ಪಡೆದುಕೊಂಡೆ........... C<br>SOUGHT ADVICE/MEDICINE FROM QUACKS/ ನಾಟಿ ವೈದ್ಯರಿಂದ ಸಲಹೆ/ಔಷದಿ ಪಡೆದುಕೊಂಡೆ .... D<br>TOOK MEDICINE AVAILABLE AT HOME/ ಮನೆಯಲ್ಲೆ ಔಷಧಿ ತೆಗೆದುಕೊಂಡೆ .. E<br>PURCHASED MEDICINE FROM SHOP WITHOUT DOCTOR'S CONSULTATION ವೈದ್ಯರ ಸಲಹೆಯಿಲ್ಲದೇ, ಅಂಗಡಿಯಿಂದ ಔಷದಿ ಖರೀದಿಸಿದೆ .... F<br>DID NOTHING/ಏನು ಮಾಡಲಿಲ್ಲಾ ..... G<br>OTHER/ ಇತರೆ_____________________97<br>(SPECIFY)	

NO.	QUESTIONS	CODING CATEGORIES	SKIP
804	How often you go for regular health check-ups?<br>ಎಷ್ಟು ದಿನಗಳಿಗೊಮ್ಮೆ ನೀವು ನಿಯಮಿತ ಆರೋಗ್ಯ ಚಿಕಿತ್ಸೆಗಾಗಿ ಭೇಟಿ ನೀಡುತ್ತೀರಿ	AT LEAST ONCE A MOTNTH ..... 1<br>ಕನಿಷ್ಟ ತಿಂಗಳಿಗೆ ಒಂದುಬಾರಿ<br>AT LEAST ONCE IN 3 MONTH.... 2<br>ಕನಿಷ್ಟ ೩ ತಿಂಗಳಿಗೆ ಒಂದುಬಾರಿ<br>WHENEVER FACED ANY PROBLEM.. 3<br>ಸಮಸ್ಯೆಯಾದಾಗ<br>RARELY/ ಅಪರೂಪ .... 4<br>CAN'T SAY/ ಹೇಳಲು ಸಾಧ್ಯವಿಲ್ಲಾ..... 8	
805	Are you currently taking any medicine for your health problem?<br>ನೀವು ನಿಮ್ಮ ಆರೋಗ್ಯ ಸಮಸ್ಯೆಗಾಗಿ ಸಧ್ಯ ಯಾವುದಾದರು ಔಷದೋಪಚಾರ ಪಡೆಯುತ್ತಿರುವಿರ?	YES/ಹೌದು .... 1<br>NO/ಇಲ್ಲಾ 0	 807
806	How long have you been using these medicines?<br>ಈ ಔಷಧವನ್ನು ಎಷ್ಟು ಸಮಯದಿಂದ ಸೇವಿಸುತ್ತಿರುವಿರಿ?<br>**RECORD "00" IF LESS THAN A MONTH**<br>**ಒಂದು ತಿಂಗಳಿಗಿಂತ ಕಡಿಮೆ ಇದ್ದಾರೆ "೦೦" ಎಂದು ದಾಖಲಿಸಿ**	RECORD DURATION IN MONTHS [ ][ ]<br>ಕಾಲಾವಧಿಯನ್ನ ತಿಂಗಳುಗಳಲ್ಲಿ ದಾಖಲಿಸಿ<br>DON'T REMEMBER/ನೆನಪಿಲ್ಲಾ...... 98	

Check Question 306: If Answer "1" GO TO Question 807 otherwise SKIP to Question 810
ಪ್ರಶ್ನೆ ಸಂಖ್ಯೆ 306 ನ್ನು ಪರಿಶೀಲಿಸಿ ಮತ್ತು ಉತ್ತರ 1 ಎಂದಿದ್ದರೆ ಪ್ರಶ್ನೆ ಸಂಖ್ಯೆ 807 ಕ್ಕೆ ಹೋಗಿ ಇಲ್ಲದಿದ್ದರೆ ಪ್ರಶ್ನೆ ಸಂಖ್ಯೆ 810 ಕ್ಕೆ ಹೋಗಿ

NO.	QUESTIONS	CODING CATEGORIES	SKIP
807	Did you face any health related problem after nirvana?<br>ನಿರ್ವಾಣ ನಂತರ ನೀವು ಯಾವುದೆ ಆರೋಗ್ಯ ಸಮಸ್ಯೆ ಎದುರಿಸಿರುವಿರಾ?<br>**[Ask this question to nirvana hijra only] CHECK WITH Q401 FOR IDENTITY**<br>**(**ಈ ಪ್ರಶ್ನೆಯನ್ನು ಕೇವಲ ನಿರ್ವಾಣ ಹಿಜಿಡಾಗಳಿಗೆ ಮಾತ್ರ ಕೇಳಿ**)**<br>ಗುರುತಿಸಲು ಪ್ರಶ್ನೆ ೪೦೧ ರೊಂದಿಗೆ ಪರೀಕ್ಷಿಸಿ.	YES/ಹೌದು .... 1<br>NO/ಇಲ್ಲಾ .... 0<br>NO ANSWER/ಪ್ರತಿಕ್ರೀಯೇ ಇಲ್ಲಾ 99	 901
808	What kind of health issues did you have as a result of nirvana?<br>ನಿರ್ವಾಣದ ಪರಿಣಾಮವಾಗಿ ಯಾವ ರೀತಿಯ ಆರೋಗ್ಯ ತೊಂದರೆಗಳನ್ನ ಅನುಭವಿಸಿರಿವಿರಿ?	PROBLEM WHILE URINATING / ಮೂತ್ರ ವಿಸರ್ಜಿಸುವಾಗ ಉರಿ .... 1<br>URINAL TRACT INFECTION / ಜನನಾಂಗದ ಸೋಂಕು .... 2<br>EXCESSIVE BLEEDING/ ಅತಿಯಾದ ರಕ್ತಸ್ರಾವ .... 3<br>INFECTION AND PUS FORMATION<br>DUE TO OPEN WOUND/ ತೆರೆದ ಗಾಯದಿಂದ ಸೊಂಕು ಮತ್ತು ಕೀವು ತುಂಬಿರುವುದು ...... 4<br>OTHER/ ಇತರೆ_________________97<br>(SPECIFY)<br>NO ANSWER/ಪ್ರತಿಕ್ರೀಯೇ ಇಲ್ಲಾ 99	
809	Did you seek any treatment for this problem?<br>ಈ ಸಮಸ್ಯೆಗೆ ನೀವು ಯಾವುದಾದರು ಚಿಕಿತ್ಸೆ ಪಡೆದಿರುವಿರ?<br>**[MULTIPLE RESPONSES ARE POSSIBLE]**<br>**[ಒಂದಕ್ಕಿಂತ ಹೆಚ್ಚು ಪ್ರತಿಕ್ರೀಯೇಗಳು ಸಾಧ್ಯವಿದೆ]**	SOUGHT ADVIC/EMEDICINE FROM CBO/NGO FACILITY ಸಿ.ಬಿ.ಒ/ಎನ್.ಜಿ.ಒ ದಿಂದ ಸಲಹೆ/ಔಷದಿ ಪಡೆದುಕೊಂಡೆ ...... A<br>SOUGHT ADVICE/MEDICINE FROM GOVERNMENT FACILITY/ ಸರ್ಕಾರಿ ಸೌಲತ್ತಿನಿಂದ ಸಲಹೆ/ಔಷದಿ ಪಡೆದುಕೊಂಡೆ .. B<br>SOUGHT ADVICE/MEDICINE FROM PRIVATE FACILITY/ ಖಾಸಗಿ ಸೌತತ್ತಿನಿಂದ ಸಲಹೆ/ಔಷದಿ ಪಡೆದುಕೊಂಡೆ .... C<br>SOUGHT ADVICEMEDICINE/ FROM QUACKS/ ನಾಟಿ ವೈದ್ಯರಿಂದ ಸಲಹೆ/ಔಷದಿ ಪಡೆದುಕೊಂಡೆ .... D<br>TOOK MEDICINE AVAILABLE AT	

		HOME/ ಮನೆಯಲ್ಲೆ ಔಷಧಿ ತೆಗೆದುಕೊಂಡೆ .. E<br>PURCHASED MEDICINE FROM SHOP WITHOUT DOCTOR'S CONSULTATION ವೈದ್ಯರ ಸಲಹೆಯಿಲ್ಲದೇ, ಅಂಗಡಿಯಿಂದ ಔಷದಿ ಖರೀದಿಸಿದೆ .... F<br>DID NOTHING/ಏನು ಮಾಡಲಿಲ್ಲಾ ..... G<br>OTHER/ ಇತರೆ_____________________97<br>(SPECIFY)	
810	Have you ever taken any hormonal treatment?<br>ನೀವು ಯಾವಾಗಲಾದರು ಹಾರ್ಮೋನಲ್ ಚಿಕಿತ್ಸೆ ಪಡೆದಿರುವಿರಾ?	YES/ಹೌದು .... 1<br>NO/ಇಲ್ಲಾ .... 0<br>NO ANSWER/ಪ್ರತಿಕ್ರೀಯೇ ಇಲ್ಲಾ 99	901
811	How long you have been taking hormonal treatement<br>ಹಾರ್ಮೋನಲ್ ಚಿಕಿತ್ಸೆಯನ್ನು ಎಷ್ಟು ಅವಧಿಯಿಂದ ತೆಗೆದುಕೊಳ್ಳುತ್ತಿರುವಿರಿ	No.of MONTHS/ ತಿಂಗಳು .... ☐☐	

SECTION IX: ANXIETY AND DEPRESSION

ವಿಭಾಗ ೯: ಆತಂಕ ಮತ್ತು ಖಿನ್ನತೆ

NO.	QUESTIONS	CODING CATEGORIES				SKIP
	**A.ANXIETY/ಆತಂಕ**					
		**NEVER** ಯಾವಾಗಲು ಇಲ್ಲಾ	**SOME-TIMES** ಕೆಲವು ಸಮಯ	**ALWAYS** ಯಾವಾ-ಗಲು ಇದೆ	**NA** ಪ್ರತಿಕ್ರೀಯೇ ಇಲ್ಲಾ	
901	Do you constantly worry about things?<br>ಕೆಲವು ಸಾಮಾನ್ಯ ವಿಷಯಗಳ ಬಗ್ಗೆ ನೀವು ಆಗಾಗ್ಗೆ ಚಿಂತೆಗೊಳಗಾಗುತ್ತೀರಾ?	0	1	2	9	
902	Do you anticipate the worst in any situation?<br>ಯಾವುದಾದರೂ ಸಂದರ್ಭಗಳಲ್ಲಿ ನೀವು ಕೆಟ್ಟದ್ದನ್ನು ನಿರೀಕ್ಷಿಸುತ್ತೀರಾ?	0	1	2	9	
	**B.DEPRESSION/ ಖಿನ್ನತೆ**					
903	Do you feel low or sad?<br>ನೀವು ಮಾನಸಿಕ ನೋವು ಅಥವ ದುಃಖಕ್ಕೆ ಒಳಗಾಗುತ್ತಿದ್ದಿರ?	0	1	2	9	
904	Do you lose interest in daily activities?<br>ದಿನನಿತ್ಯದ ಕೆಲಸದಲ್ಲಿ ಆಸಕ್ತಿ ಕಳೆದುಕೊಳ್ಳುತ್ತೀರಾ?	0	1	2	9	
905	Do you lack energy and strength?<br>ನಿಮ್ಮ ಶಕ್ತಿ-ಸಾಮರ್ಥ್ಯ ಕಡಿಮೆಯಿದೆಯಾ?	0	1	2	9	
906	Do you feel less self-confident?<br>ಆತ್ಮ ವಿಶ್ವಾಸ ಕಳೆದುಕೊಂಡಂತೆ ಅನಿಸುತ್ತಿದೆಯಾ?	0	1	2	9	
907	Do you feel that life isn't worth living? ಜೀವನ ಬದುಕಲು ಯೋಗ್ಯವಲ್ಲ ಎಂದೆನ್ನಿಸುತ್ತದೆಯೇ?	0	1	2	9	
908	Do you have difficulty in concentrating on day to day activities?<br>ದಿನನಿತ್ಯದ ಕೆಲಸ ಕಾರ್ಯಗಳನ್ನು ನಿರ್ವಹಿಸಲು ನಿಮಗೆ ಕಷ್ಟ ಎಂದೆನ್ನಿಸುತ್ತದೆಯೇ?	0	1	2	9	
909	Do you have trouble sleeping at night?<br>ನಿಮಗೆ ರಾತ್ರಿ ನಿದ್ರೆ ಮಾಡಲು ತೊಂದರೆಯಾಗುತ್ತದೆಯೇ?	0	1	2	9	
910	Do you feel changes in your appetite?<br>ನಿಮ್ಮ ಹಸಿವಿನಲ್ಲಿ ವ್ಯತ್ಯಾಸವಿದೆಯಾ?	0	1	2	9	

SECTION X: SUICIDALITY
ವಿಭಾಗ ೧೦: ಆತ್ಮಹತ್ಯೆ

NO.	QUESTIONS	CODING CATEGORIES	SKIP
1001	In the past month did you:<br>ಕಳೆದ ತಿಂಗಳಿನಲ್ಲಿ ನಿಮಗೆ ಈ ಅನುಭವಗಳು ಆಗಿವೆಯೇ:		
	A. Think that you would be better off dead or wish you were dead?<br>ಎ. ಸಾಯುವುದು ಒಳ್ಳೆಯುವುದು ಎಂದು ಯೋಚಿಸಿದ್ದಿರಾ?	YES/ಹೌದು .... 1<br>NO/ಇಲ್ಲಾ .... 0<br>NO ANSWER/ಪ್ರತಿಕ್ರೀಯೇ ಇಲ್ಲಾ 99	
	B. Want to harm/injure yourself?<br>ಬಿ. ನಿಮಗೆ ನೀವೆ ಹಿಂಸಿಸಿಕೊಂಡಿರುವಿರಾ?	YES/ಹೌದು .... 1<br>NO/ಇಲ್ಲಾ .... 0<br>NO ANSWER/ಪ್ರತಿಕ್ರೀಯೇ ಇಲ್ಲಾ 99	
	C. Thought about suicide<br>ಸಿ. ಆತ್ಮಹತ್ಯೆ ಮಾಡಿಕೊಳ್ಳಲು ಯೋಚಿಸಿರುವಿರಾ?	YES/ಹೌದು .... 1<br>NO/ಇಲ್ಲಾ .... 0<br>NO ANSWER/ಪ್ರತಿಕ್ರೀಯೇ ಇಲ್ಲಾ 99	
1002	Have you deliberately tried to injure yourself?<br>ಸಧ್ಯ ನೀವು ನಿಮ್ಮನ್ನು ಉದ್ದೇಶಪೂರ್ವಕವಾಗಿ ಗಾಯಮಾಡಿಕೊಳ್ಳಲು ಪ್ರಯತ್ನಿಸಿರುವಿರಾ?	YES/ಹೌದು .... 1<br>NO/ಇಲ್ಲಾ .... 0<br>NO ANSWER/ಪ್ರತಿಕ್ರೀಯೇ ಇಲ್ಲಾ 99	
1003	Have you ever made a suicide attempt in your life?<br>ನಿಮ್ಮ ಜೀವನದಲ್ಲಿ ಎಂದಾದರು ಆತ್ಮಹತ್ಯೆಗೆ ಪ್ರಯತ್ನ ಮಾಡಿರುವಿರಾ?	YES/ಹೌದು .... 1<br>NO/ಇಲ್ಲಾ .... 0<br>NO ANSWER/ಪ್ರತಿಕ್ರೀಯೇ ಇಲ್ಲಾ 99	1101 (NO, NO ANSWER)
1004	In past one month have you tried to commit suicide or harm yourself?<br>ಕಳೆದ ಒಂದು ತಿಂಗಳಿನಲ್ಲಿ ಆತ್ಮಹತ್ಯೆ ಅಥವ ನಿಮ್ಮನ್ನು ನೀವು ಹಿಂಸಿಸಿಕೋಳ್ಳಲು ಪ್ರಯತ್ನಿಸಿರುವಿರಾ?	YES/ಹೌದು .... 1<br>NO/ಇಲ್ಲಾ .... 0<br>NO ANSWER/ಪ್ರತಿಕ್ರೀಯೇ ಇಲ್ಲಾ 99	

SECTION XI: VIOLENCE
ವಿಭಾಗ XI: ಹಿಂಸೆ

NO.	QUESTIONS	CODING CATEGORIES	SKIP
1101	In last six months, how many times would you say someone has beaten (hurt, hit, slapped, pushed, kicked, punched, choked or burned, but not used weapon) you?<br>ಕಳೆದ 6 ತಿಂಗಳಲ್ಲಿ ಎಷ್ಟು ಬಾರಿ ಜನ ನಿಮ್ಮನ್ನು ಹೊಡೆದಿದ್ದಾರೆ (ಯಾವುದೆ ಅಸ್ತ್ರಗಳನ್ನ ಬಳಸದೆ ನೋಯಿಸುವುದು, ಹೊಡೆಯುವುದು, ತಳ್ಳುವುದು, ಒದೆಯುವುದು, ಗುದ್ದೋದು, ಕುತ್ತಿಗೆ ಹಿಸುಕುವುದು, ಸುಡುವುದು)	ONCE/ಒಮ್ಮೆ .... 01<br>2-5 TIMES/2-5 ಬಾರಿ .... 02<br>6-10 TIMES OR MORE .... 03<br>6-10 ಬಾರಿ ಅಥಾ ಹೆಚ್ಚು<br>NEVER/ಒಮ್ಮೆಯು ಇಲ್ಲಾ.......... 00<br>NO ANSWER/ಪ್ರತಿಕ್ರೀಯೇ ಇಲ್ಲಾ 99	1104 (NEVER, NO ANSWER)
1102	Who did this to you?<br>ನಿಮಗೆ ಇದನ್ನು ಯಾರು ಮಾಡಿದರು<br>**[MULTIPLE RESPONSES ARE POSSIBLE]**<br>**[ಒಂದಕ್ಕಿಂತ ಹೆಚ್ಚು ಪ್ರತಿಕ್ರೀಯೇಗಳು ಸಾಧ್ಯವಿದೆ]**	PANTHI/ಪಂಥಿ................. A<br>GURU/ಗುರು.................... B<br>GOON/ಗುಂಡ .... C<br>POLICE/ಪೋಲಿಸ................ D<br>OTHER/ ಇತರೆ____________________97<br>(SPECIFY)<br>NO ANSWER/ಪ್ರತಿಕ್ರೀಯೇ ಇಲ್ಲಾ 99	1104 (C–99)
1103	Now I'd like to ask you some questions about things that happened with you<br>ಈಗ ನಾನು ನಿಮಗೆ ಆದ ಕೆಲವು ಅನುಭವಗಳು/ಘಟನೆಗಳ ಬಗ್ಗೆ ಕೆಲವು ಪ್ರಶ್ನೆಗಳನ್ನು ಕೇಳಲು ಬಯಸುತ್ತೇನೆ<br><br>A. Has your Guru/Panthi/partner<br>ನಿಮ್ಮ ಗುರು/ಪಂಥಿ ಅಥವ ಪಾರ್ಟನರ್<br><br>a) Said or done something to humiliate you in front of others? ಇತರರ ಮುಂದೆ ನಿಮ್ಮನ್ನು ಹೀನೈಸುವ ಮಾತುಗಳನ್ನಾಡಿದ್ದಾರೆಯೇ ಅಥವಾ ಹೀನೈಸುವಂತೆ ನಡೆದುಕೊಂಡಿದ್ದಾರೆಯೇ?	B. How often has this happened during the last 6 months: often, only sometimes, or not at all?<br>ಕಳೆದ 6 ತಿಂಗಳಲ್ಲಿ ಈ ರೀತಿಯಾಗಿ ಎಷ್ಟು ಬಾರಿ ಸಂಭವಿಸಿತ್ತು ? ಅನೇಕ ಬಾರಿ ಹೆಚ್ಚಾಗಿ. ಕೆಲವೊಮ್ಮೆ, ಎಂದೂ ಇಲ್ಲ<br><br>MANY TIMES ಅನೇಕ ಬಾರಿ / A FEW TIMES ಕೆಲವೊಮ್ಮೆ / NOT AT AT ALL ಎಂದೂ ಇಲ್ಲ<br><br>YES.....1 → 1 2 3<br>NO......0	

	b) Threatened to hurt or harm you or someone close to you? ನಿಮ್ಮನ್ನು ಅಥವಾ ನಿಮ್ಮ ಹತ್ತಿರದವರನ್ನು ನೋಯಿಸುವುದಾಗಿ ಅಥವಾ ತೊಂದರೆಮಾಡುವುದಾಗಿ ಬೆದರಿಸಿದ್ದಾರೆಯೇ?<br>c) Insulted you repeatedly to make you feel bad about yourself? ನಿಮ್ಮ ಬಗ್ಗೆ ನಿಮಗೆ ತಿರಸ್ಕಾರ ಬರುವಂತೆ ನಿಮ್ಮನ್ನು ಪದೇ ಪದೇ ಅವಮಾನ ಮಾಡಿದ್ದಾರೆಯೇ?	YES.....1 → 1 2 3<br>NO......0<br><br>YES.....1 → 1 2 3<br>NO......0	
1104	In the past one-year, were you ever beaten or otherwise physically forced to have sexual intercourse with someone even though you didn't want to?<br>ಕಳೆದ ಒಂದು ವರ್ಷದಲ್ಲಿ ಯಾವಾಗಲಾದರು ಯಾರಾದರು ನಿಮ್ಮನ್ನು ಹೊಡೆದಿದ್ದಾರೆಯೇ ಅಥವ ನಿಮ್ಮ ಇಚ್ಚೆಗೆ ವಿರುದ್ದವಾಗಿ ನಿಮ್ಮನ್ನು ಲೈಂಗಿಕ ಸಂಪರ್ಕಕ್ಕೆ ಬಲವಂತ ಮಾಡಿದ್ದಾರೆಯೇ?	NO/ಇಲ್ಲಾ....................00<br>YES/ಹೌದು.................. 01<br>NOT APPLICABLE/ಅನ್ವಯಿಸುವುದಿಲ್ಲಾ. 97<br>NO ANSWER/ಪ್ರತಿಕ್ರೀಯೇ ಇಲ್ಲಾ...... 99	
1105	Do you know of crisis helplines and contacts of people you can call if you are in a situation where violence might occur?<br>ಯಾವುದೆ ಹಿಂಸೆ ಆದಾಗ ಸಂಪರ್ಕಿಸಬಹುದಾದ ಸಹಾಯವಾಣಿ ಅಥವ ಸಂಬಂಧಿಸಿದ ವ್ಯಕ್ತಿಗಳ ಫೋನ್ ನಂಬರಗಳು ನಿಮಗೆ ತಿಳಿದಿದೆಯೇ?	NO/ಇಲ್ಲಾ....................00<br>YES/ಹೌದು.................. 01<br>NO ANSWER/ಪ್ರತಿಕ್ರೀಯೇ ಇಲ್ಲಾ...... 99	→1201
1106	Did you informed to crisis management team about the violence you faced in last six months?<br>ಕಳೆದ 6 ತಿಂಗಳಿನಲ್ಲಿ ನೀವು ಅನುಭವಿಸಿದ ಯಾವುದೆ ಹಿಂಸೆಯನ್ನು ಬಿಕ್ಕಟ್ಟು ನಿರ್ವಹಣಾ ತಂಡದ ಗಮನಕ್ಕೆ ತಂದಿರುವಿರಾ?	NO/ಇಲ್ಲಾ....................00<br>YES/ಹೌದು.................. 01<br>DID NOT FACE VIOLENCE......97<br>ಹಿಂಸೆ ಅನುಭವಿಸಿಲ್ಲಾ<br>NO ANSWER/ಪ್ರತಿಕ್ರೀಯೇ ಇಲ್ಲಾ...... 99	→ 1108<br><br><br>→ 1201
1107	Did crisis management team address the issue?<br>ಬಿಕ್ಕಟ್ಟು ನಿರ್ವಹಣಾ ತಂಡವು ನಿಮ್ಮ ಸಮಸ್ಯೆಯನ್ನು ಬಗೆಹರಿಸಿತೆ?	YES, WITHIN 24 HRS/<br>ಹೌದು, 24 ಗಂಟೆಗಳ ಅವಧಿಯಲ್ಲಿ ....01<br>YES, WITHIN A MONTH/ ಹೌದು, ತಿಂಗಳ ಅವಧಿಯಲ್ಲಿ ....02<br>NOT YET ADDRESSED /<br>ಇನ್ನೂ ಬಗೆಹರಿಸಿಲ್ಲ.......... 00<br>NO ANSWER/ಪ್ರತಿಕ್ರೀಯೇ ಇಲ್ಲಾ...... 99	1201
1108	If no, why didn't you report the incident?<br>ಇಲ್ಲಾವೆಂದಾದಲ್ಲಿ, ಯಾಕೆ ದೂರನ್ನು ಹೇಳಲಿಲ್ಲಾ	DUE TO FEAR/ ಭಯದ ಕಾರಣದಿಂದ...01<br>DUE TO SHAME/ ನಾಚಿಕೆಯಿಂದ ... 02<br>DUE TO THREAT TO LIFE/ ಜೀವ ಭಯದಿಂದ .... 03<br>DUE TO GUILT/ ಅಪರಾದ ಮನೋಭಾವದಿಂದ .... 04<br>OTHER/ ಇತರೆ______________________97<br>NO ANSWER/ಪ್ರತಿಕ್ರೀಯೇ ಇಲ್ಲಾ 99	

SECTION XII: COPING STRATEGIES
ವಿಭಾಗ XII: ನಿಭಾಯಿಸುವ ತಂತ್ರಗಾರಿಕೆ

I would like to ask you some questions on managing the stress and difficult situation you faced.
ಒತ್ತಡ ನಿರ್ವಹಣೆ ಮತ್ತು ನೀವು ಎದುರಿಸಿದ ಸಂಕಷ್ಟ ಪರಿಸ್ಥಿತಿಯ ಕುರಿತು ನಾನೀಗ ಕೆಲವು ಪ್ರಶ್ನೆಗಳನ್ನ ಕೇಳಲಿಚ್ಚಿಸುತ್ತೇನೆ.

NO.	QUESTIONS	CODING CATEGORIES				SKIP
1201	When you are in a difficult situation what do you do?<br>ನೀವು ಸಂಕಷ್ಟದ ಸನ್ನಿವೇಶದಲ್ಲಿ ಇದ್ದಾಗ ಏನು ಮಾಡುವಿರಿ?	**NEVER** ಯಾವಾಗಲು ಇಲ್ಲಾ	**SOME-TIMES** ಕೆಲವು ಸಮಯ	**ALWAYS** ಯಾವಾ-ಗಲು ಇದೆ	**NA** ಪ್ರತಿಕ್ರೀಯೇ ಇಲ್ಲಾ	
	A. Check with friends and community members on what to do<br>ಎ. ಏನು ಮಾಡುವುದು ಎಂದು ಸ್ನೆಹಿತರು ಮತ್ತು ಸಮುದಾಯ ಸದಸ್ಯರಲ್ಲಿ ವಿಚಾರಿಸಿರುವುದು.	0	1	2	9	

NO.	QUESTIONS	CODING CATEGORIES				SKIP
		NEVER<br>ಯಾವಾಗಲು ಇಲ್ಲಾ	SOME-TIMES<br>ಕೆಲವು ಸಮಯ	ALWAYS<br>ಯಾವಾ-ಗಲು ಇದೆ	NA<br>ಪ್ರತಿಕ್ರೀಯೇ ಇಲ್ಲಾ	
	B. Depend on myself and my personal strengths<br>ಬಿ. ನನ್ನ ವೈಯಕ್ತಿಕ ಶಕ್ತಿಯ ಮೇಲೆ ನಂಬಿಕೆ ಇದೆ	0	1	2	9	
	C. Avoid dealing with particular problem<br>ಸಿ. ಸಮಸ್ಯೆಗಳಿಂದ ತಪ್ಪಿಸಿಕೊಳ್ಳುವುದು	0	1	2	9	
	D. Get engaged in other activities to avoid thinking about the problem<br>ಡಿ. ಆ ಸಮಸ್ಯೆ ಬಗ್ಗೆ ಯೋಚಿಸುವುದರಿಂದ ಹೊರಗುಳಿಯಲು ಬೇರೆಕೆಲಸ ಕಾರ್ಯಗಳಲ್ಲಿ ತೊಡಗಿಸಿಕೊಳ್ಳೂವುದು.	0	1	2	9	
	E. Drink alcohol to feel better<br>ಈ. ಸಮಾಧಾನಕ್ಕಾಗಿ ಮಧ್ಯಪಾನ ಮಾಡುವುದು	0	1	2	9	
	F. Criticize myself for letting the problem happen<br>ಎಪ್. ನನ ಬಗ್ಗೆ ಕೀಳರಿಮೇ.	0	1	2	9	
	G. Make jokes about it to lighten the issue.<br>ಜಿ. ಆ ವಿಷಯದ ಬಗ್ಗೆ ಹಾಸ್ಯ ಮಾಡುವುದು	0	1	2	9	

SECTION XIII: EXPOSURE TO INTERVENTION
ವಿಭಾಗ XIII:ಇಂಟರ್‌ವೆನ್‌ಶನ್ ಎಕ್ಸ್‌ಪೋಸರ್

Now I would like to ask you a few questions regarding the HIV prevention program among MSM in Bangalore.
ಈಗ ನಾನು ಬೆಂಗಳೂರಿನಲ್ಲಿ ಎಮ್‌ಎಸ್‌ಎಂಗಳಿಗಾಗಿ ಇರುವ ಹೆಚ್.ಐ.ವಿ ತಡೆಗಟ್ಟುವ ಕಾರ್ಯಕ್ರಮದ ಬಗ್ಗೆ ಕೆಲವು ಪ್ರಶ್ನೆಗಳನ್ನ ಕೇಳುತ್ತೇನೆ.

NO.	QUESTIONS	CODING CATEGORIES	SKIP
1301	Since how long have you been aware of the SANGAMA/SAMARA programs for prevention of HIV among MSM-T in Bangalore?<br>ಬೆಂಗಳೂರಿನಲ್ಲಿ MSM-T ಗಳೋಂದಿಗೆ ಸಂಗಮ/ಸಮರ ನಡೆಸುತ್ತಿರುವ ಹೆಚ.ಐ.ವಿ ತಡೆಗಟ್ಟುವ ಕಾರ್ಯಕ್ರಮದ ಬಗ್ಗೆ ನಿಮಗೆ ಎಷ್ಟು ದಿನಗಳಿಂದ ತಿಳಿದಿದೆ.<br><br>IF <1 WEEK, RECORD IN DAYS<br>ಒಂದು ವಾರಕ್ಕಿಂತ ಕಡಿಮೆ ಇದ್ದರೆ, ದಿನಗಳಲ್ಲಿ ದಾಖಲಿಸಿ<br>IF >1 WEEK AND <1 MONTH, RECORD IN WEEKS<br>ಒಂದು ವಾರಕ್ಕಿಂತ ಅಧಿಕ ಮತ್ತು ಒಂದು ತಿಂಗಳಿಗಿಂತ ಕಡಿಮೆ ಇದ್ದರೆ, ವಾರಗಳಲ್ಲಿ ದಾಖಲಿಸಿ<br>IF >1 MONTH AND <1 YEAR, RECORD IN MONTHS<br>ಒಂದು ತಿಂಗಳಿಗಿಂತ ಅಧಿಕ ಮತ್ತು ಒಂದು ವರ್ಷಕ್ಕಿಂತ ಕಡಿಮೆ ಇದ್ದರೆ, ತಿಂಗಳಿನಲ್ಲಿ ದಾಖಲಿಸಿ<br>IF >1 YEAR, RECORD IN YEARS<br>ಒಂದು ವರ್ಷಕ್ಕಿಂತ ಅಧಿಕವಾಗಿದ್ದರೆ, ವರ್ಷಗಳಲ್ಲಿ ದಾಖಲಿಸಿ	DAYS/ದಿನಗಳು .... 1 [ ][ ]<br>WEEKS/ವಾರಗಳು .... 2 [ ][ ]<br>MONTHS/ತಿಂಗಳು .... 3 [ ][ ]<br>YEARS/ವರ್ಷ .... 4 [ ][ ]<br><br>DON'T KNOW/ಗೊತ್ತಿಲ್ಲಾ.............. 98<br>NO ANSWER/ಪ್ರತಿಕ್ರೀಯೇ ಇಲ್ಲಾ......... 99	
1302	Have you been contacted by a peer educator/outreach worker from the CBO program in the last one month?<br>ಕಳೆದ ಒಂದು ತಿಂಗಳಿನಲ್ಲಿ ಎ.ಜಿ.ಓ ಕಾರ್ಯಕ್ರಮದ ಪೀಯರ್ ಅಥವ ಔಟರೀಚ್ ವರ್ಕರ್‌ಗಳು ನಿಮ್ಮನ್ನು ಸಂಪರ್ಕಿಸಿದ್ದಾರೆಯೇ?	NO/ಇಲ್ಲಾ.......................00<br>YES/ಹೌದು......................01<br>NO ANSWER/ಪ್ರತಿಕ್ರೀಯೇ ಇಲ್ಲಾ..........99	
1303	Have you visited the clinic run by the CBO program in last 3 months?<br>ಕಳೆದ ಮೂರು ತಿಂಗಳಿನಲ್ಲಿ ನೀವು ಎನ್.ಜಿ.ಓ ಕಾರ್ಯಕ್ರಮದಲ್ಲಿ ನಡೆಸುತ್ತಿರುವ ಕ್ಲೀನಿಕ್ ಗಳಿಗೆ ಬೇಟ್ಟಿ ನೀಡಿದ್ದೀರಾ?	NO/ಇಲ್ಲಾ.......................00<br>YES/ಹೌದು......................01<br>NO ANSWER/ಪ್ರತಿಕ್ರೀಯೇ ಇಲ್ಲಾ.........99	

NO.	QUESTIONS	CODING CATEGORIES	SKIP
1304	Have you received counseling services from the project in last one month<br>ಕಳೆದ ಒಂದು ತಿಂಗಳ ಅವಧಿಯಲ್ಲಿ ನೀವು ಯೋಜನಾ-ಕಾರ್ಯಕ್ರಮಗಳ ವತಿಯಿಂದ ಸಮಾಲೋಚನಾ ಸೇವೆಯನ್ನ ಪಡೆದಿರುವಿರಾ?	NO/ಇಲ್ಲಾ........................00<br>YES/ಹೌದು.......................01<br>NO ANSWER/ಪ್ರತಿಕ್ರೀಯೇ ಇಲ್ಲಾ..........99	
1305	Have you taken part in any meeting/training organized by the CBO in last one month<br>ಕಳೆದ ಒಂದು ತಿಂಗಳಿನಲ್ಲಿ ಎನ್.ಜಿ.ಒ ಸಂಘಟಿಸಿದ್ದ ಯಾವುದಾದರು ಮೀಟಿಂಗ್/ಟ್ರೈನಿಂಗಗಳಲ್ಲಿ ಭಾಗವಹಿಸಿರುವಿರಾ?	NO/ಇಲ್ಲಾ........................00<br>YES/ಹೌದು.......................01<br>NO ANSWER/ಪ್ರತಿಕ್ರೀಯೇ ಇಲ್ಲಾ..........99	
1306	Are you a member of any community-based organization of MSM TMSW self-help group?<br>ನೀವು ಯಾವುದಾದರು MSM TMSW ಸಮುದಾಯ ಆದಾರಿತ ಸಂಘಟನೆಯ ಸ್ವಸಹಾಯ ಸಂಘದ ಸದಸ್ಯರಾಗಿರುವಿರಾ?<br>[A formal group comprised of and managed by MSM-T/MSW members]<br>(MSM-T/MSW ಗಳನ್ನ ಒಳಗೊಂಡ ಮತ್ತು ಅವರಿಂದಲೆ ನಿರ್ವಹಿಸಲ್ಪಡುತ್ತಿರುವ ಗುಂಪು)	NO/ಇಲ್ಲಾ........................00<br>YES/ಹೌದು...................... 0<br>DON'T KNOW/REMEMBER/ನೆನಪಿಲ್ಲಾ.....98<br>NO ANSWER/ಪ್ರತಿಕ್ರೀಯೇ ಇಲ್ಲಾ.........99	→1309 (NO)<br>1309 (98, 99)
1307	Why did you join this group?<br>ನೀವು ಯಾಕೆ ಈ ಸಂಘಟನೆಯನ್ನ ಸೇರಿರುವಿರಿ?<br>DO NOT READ RESPONSES/ಪ್ರತಿಕ್ರೀಯೆಗಳನ್ನ ಓದಬೇಡಿ	THE GROUP PROVIDES USEFUL SERVICES FOR MY COMMUNITY/ ಗುಂಪು ನನ್ನ ಸಮುದಾಯಕ್ಕೆ ಒಳ್ಳೆ ಸೇವೆಗಲನ್ನ ವದಗಿಸುತ್ತಿದೆ .... 1<br>CBO REQUESTED ME/ಸಂಘಟನೆ ನನ್ನನ್ನು ವಿನಂತಿಸಿಕೊಂಡಿತು .... 2<br>MY OTHER FRIENDS WERE PART OF IT/ ನನ್ನ ಇತರ ಸ್ನೇಹಿತರು ಇದರಲ್ಲಿ ಪಾಲ್ಗೊಂಡಿದ್ದಾರೆ . 3<br>GROUP PROVIDES ME PERSONAL/MORAL SUPPORT/ ಗುಂಪು ನನಗೆ ವೈಯಕ್ತಿಕ/ನೈತಿಕ ಬೆಂಬಲ ನೀಡುತ್ತದೆ .... 4<br>OTHER/ ಇತರೆ________________________97<br>(SPECIFY)	
1308	How long have you been a member of this group<br>ಎಷ್ಟು ಸಮಯದಿಂದ ನೀವು ಈ ಸಂಘಟನೆಯ ಸದಸ್ಯರಾಗಿರುವಿರಿ?<br>IF <1 WEEK, RECORD IN DAYS<br>ಒಂದು ವಾರಕ್ಕಿಂತ ಕಡಿಮೆ ಇದ್ದರೆ, ದಿನಗಳಲ್ಲಿ ದಾಖಲಿಸಿ<br>IF >1 WEEK AND <1 MONTH, RECORD IN WEEKS<br>ಒಂದು ವಾರಕ್ಕಿಂತ ಅಧಿಕ ಮತ್ತು ಒಂದು ತಿಂಗಳಿಗಿಂತ ಕಡಿಮೆ ಇದ್ದರೆ, ವಾರಗಳಲ್ಲಿ ದಾಖಲಿಸಿ<br>IF >1 MONTH AND <1 YEAR, RECORD IN MONTHS<br>ಒಂದು ತಿಂಗಳಿಗಿಂತ ಅಧಿಕ ಮತ್ತು ಒಂದು ವರ್ಷಕ್ಕಿಂತ ಕಡಿಮೆ ಇದ್ದರೆ, ತಿಂಗಳಿನಲ್ಲಿ ದಾಖಲಿಸಿ<br>IF >1 YEAR, RECORD IN YEARS<br>ಒಂದು ವರ್ಷಕ್ಕಿಂತ ಅಧಿಕವಾಗಿದ್ದರೆ, ವರ್ಷಗಳಲ್ಲಿ ದಾಖಲಿಸಿ	DAYS/ದಿನಗಳು .... 1 [ ][ ]<br>WEEKS/ವಾರಗಳು .... 2 [ ][ ]<br>MONTHS/ತಿಂಗಳು .... 3 [ ][ ]<br>YEARS/ವರ್ಷ .... 4 [ ][ ]<br>DON'T KNOW/ಗೊತ್ತಿಲ್ಲಾ.............. 98<br>NO ANSWER/ಪ್ರತಿಕ್ರೀಯೇ ಇಲ್ಲಾ 99	
1309	Why are you not a member of a community group sex worker collective self-help group?<br>ಯಾಕೆ ನೀವು ಸಮುದಾಯದ ಗುಂಪು/ಲೈಂಗಿಕ ವೃತ್ತಿನಿರತರ ಸಂಘಟನೆ/ಸ್ವಸಹಾಯ ಸಂಘದ ಸದಸ್ಯರಾಗಿಲ್ಲ<br>**CIRCLE ALL THAT ARE MENTIONED**<br>**ಉತ್ತರಿಸಿದ ಎಲ್ಲಾ ಪ್ರತಿಕ್ರೀಯೇಗಳನ್ನ ದಾಖಲಿಸಿ.**	DON'T KNOW A GROUP/ ಗುಂಪಿನ ಬಗ್ಗೆ ತಿಳಿದಿಲ್ಲಾ .. A<br>SCARED TO JOIN/ ಸೇರಲು ಭಯ .... B<br>DON'T UNDERSTAND THE ADVANTAGE/ BENEFIT OF JOINING /ಸೇರುವುದರಿಂದ ಆಗುವ ಲಾಭ/ಉಪಯೋಗದ ಬಗ್ಗೆ ತಿಳಿದಿಲ್ಲಾ .... C<br>NO TIME/NOT INTERESTED......... D<br>ಸಮಯವಿಲ್ಲ/ಆಸಕ್ತಿ ಇಲ್ಲಾ<br>OTHER/ ಇತರೆ_________________________97<br>(SPECIFY)	

The interview ends here. Thank you very much for your time, and for providing the information. I assure you again that none of the information you have given us will be shared with anyone else, and your responses will remain completely anonymous.

ಇಲ್ಲಿಗೆ ಸಂದರ್ಶನ ಮುಕ್ತಾಯಗೊಂಡಿತು. ನಿಮ್ಮ ಸಮಯ ಮತ್ತು ಅಮೂಲ್ಯವಾದ ಮಾಹಿತಿ ನೀಡಿರುವುದಕ್ಕೆ ದನ್ಯವಾದಗಳು. ನಿಮ್ಮ ಪ್ರತಿಕ್ರೀಯೆಗಳು ಗೌಪ್ಯವಾಗಿರುತ್ತವೆ ಮತ್ತು ಈ ಮಾಹಿತಿಯನ್ನು ಇತರರೊಂದಿಗೆ ಹಂಚಿಕೊಳ್ಳಲಾಗುವುದಿಲ್ಲಾ ಎಂಬ ಭರವಸೆಯನ್ನು ಮತ್ತೊಮ್ಮೆ ನೀಡಲು ಇಚ್ಛಿಸುತ್ತೇನೆ.
